# Genomic characterization of two novel pathogenic avipoxviruses isolated from pacific shearwaters (*Ardenna* spp*.)*

**DOI:** 10.1186/s12864-017-3680-z

**Published:** 2017-04-13

**Authors:** Subir Sarker, Shubhagata Das, Jennifer L. Lavers, Ian Hutton, Karla Helbig, Jacob Imbery, Chris Upton, Shane R. Raidal

**Affiliations:** 1 Department of Physiology, Anatomy and Microbiology, School of Life Sciences, La Trobe University, Melbourne, VIC 3086 Australia; 2 School of Animal and Veterinary Sciences, Charles Sturt University, Wagga Wagga, NSW 2678 Australia; 3 Institute for Marine and Antarctic Studies, University of Tasmania, Hobart, TAS 7004 Australia; 4 Lord Howe Island Museum, Lord Howe Island, NSW 2898 Australia; 5 Department of Biochemistry and Microbiology, University of Victoria, Victoria, BC Canada

**Keywords:** *Avipoxvirus*, Poxvirus, Next generation sequencing, dermatitis, *Ardenna*, Shearwater

## Abstract

**Background:**

Over the past 20 years, many marine seabird populations have been gradually declining and the factors driving this ongoing deterioration are not always well understood. Avipoxvirus infections have been found in a wide range of bird species worldwide, however, very little is known about the disease ecology of avian poxviruses in seabirds. Here we present two novel avipoxviruses from pacific shearwaters (*Ardenna* spp), one from a Flesh-footed Shearwater (*A. carneipes*) (SWPV-1) and the other from a Wedge-tailed Shearwater (*A. pacificus)* (SWPV-2).

**Results:**

Epidermal pox lesions, liver, and blood samples were examined from *A. carneipes* and *A. pacificus* of breeding colonies in eastern Australia. After histopathological confirmation of the disease, PCR screening was conducted for avipoxvirus, circovirus, reticuloendotheliosis virus, and fungal agents. Two samples that were PCR positive for poxvirus were further assessed by next generation sequencing, which yielded complete *Shearwaterpox virus* (SWPV) genomes from *A. pacificus* and *A. carneipes,* both showing the highest degree of similarity with *Canarypox virus* (98% and 67%, respectively). The novel SWPV-1 complete genome from *A. carneipes* is missing 43 genes compared to CNPV and contains 4 predicted genes which are not found in any other poxvirus, whilst, SWPV-2 complete genome was deemed to be missing 18 genes compared to CNPV and a further 15 genes significantly fragmented as to probably cause them to be non-functional.

**Conclusion:**

These are the first avipoxvirus complete genome sequences that infect marine seabirds. In the comparison of SWPV-1 and −2 to existing avipoxvirus sequences, our results indicate that the SWPV complete genome from *A. carneipes* (SWPV-1) described here is not closely related to any other avipoxvirus genome isolated from avian or other natural host species, and that it likely should be considered a separate species.

**Electronic supplementary material:**

The online version of this article (doi:10.1186/s12864-017-3680-z) contains supplementary material, which is available to authorized users.

## Background

The *Avipoxvirus* genus includes a divergent group of viruses that cause diseases in more than 278 species of wild and domestic birds in terrestrial and marine environments worldwide [1, 2]. Relatively little is known about the origins, worldwide host distribution and genetic diversity of avipoxviruses [3]. In affected birds, avipoxviruses typically cause proliferative ‘wart-like’ growths that are most commonly restricted to the eyes, beak or unfeathered skin of the body (so-called ‘dry’ pox), but infections can also develop in the upper alimentary and respiratory tracts (‘wet’ or ‘diptheritic’ pox) [2]. The incubation period and magnitude of avipoxvirus infection is variable, and is rarely fatal although secondary bacterial or fungal infections are common and cause increased mortality [2]. Such conditions in naïve populations can reach a much higher prevalence with substantial fatality [4, 5].

Avipoxviruses belong to the subfamily *Chordopoxvirinae* (ChPV) of the *Poxviridae* family, which are relatively large double-stranded DNA (dsDNA) viruses that replicate in the cytoplasm of infected cells [6]. Although poxviruses have evolved to infect a wide range of host species, to date only six avipoxvirus genomes have been published; a pathogenic American strain of *Fowlpox virus* (FPVUS) [7], an attenuated European strain of *Fowlpox virus* (FP9) [8], a virulent *Canarypox virus* (CNPV) [9], a pathogenic South African strain of *Pigeonpox virus* (FeP2), a *Penguinpox virus* (PEPV) [3], and a pathogenic Hungarian strain of *Turkeypox virus* (TKPV) [10]. Although these genome sequences demonstrate that avipoxviruses have diverged considerably from the other chordopoxviruses (ChPVs), approximately 80 genes have been found to be conserved amongst all ChPVs and to comprise the minimum essential poxvirus genome [11]. These genes tend to be present in the central core of the linear genome with the remainder presumed to be immunomodulatory and host specific genes located towards the terminal regions of the genome [3]. With the exception of TKPV (188 kb), avipoxvirus genomes (266–360 kb) tend to be bigger than those of other ChPVs due in part to multiple families of genes.

Over the past two decades, the status of the world’s bird populations have deteriorated with seabirds declining faster than any other group of birds [12]. On Lord Howe Island in eastern Australia, the Flesh-footed Shearwater *Ardenna carneipes* has been declining for many years and is therefore listed as Vulnerable in the state of New South Wales [13]. The ongoing threat of plastic pollution, and toxicity from the elevated concentration of trace elements such as mercury could be confounding drivers of this declining species [14]. Infectious diseases, including those caused by avipoxviruses, have also been identified as an important risk factor in the conservation of small and endangered populations, particularly in island species [15–18]. The impact of the introduction of avipoxviruses has been severe for the avifauna of various archipelagos [19]. The emergence of distinctive avipoxvirus with a high prevalence (88%) in Hawaiian Laysan Albatross (*Phoebastria immutabilis*) enabled one of the first detailed studies of the epidemiology and population-level impact of the disease in the seabirds [20]. However, relatively little is known about the general prevalence or effects of poxviruses in seabird species, including for shearwaters (*Ardenna* or *Puffinus* spp.). Therefore, the aim of the present study was to identify and characterize pathogens associated with clinical disease in breeding colonies of Flesh-footed Shearwater and Wedge-tailed Shearwater sourced from Lord Howe Island in 2015.

## Results

### Identification of fungal pathogens

**Fig. 1:**
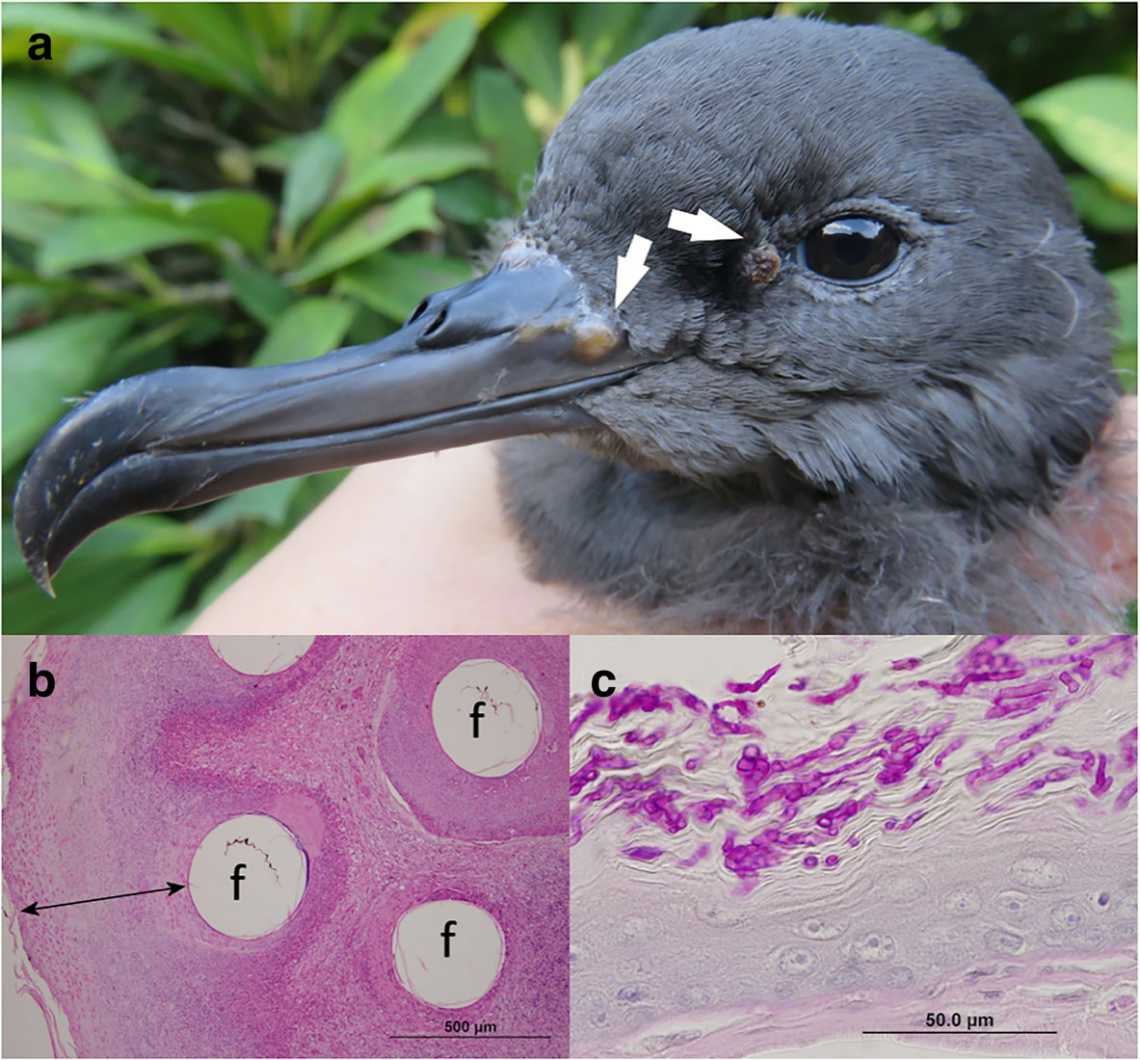
Pathological evidence of characteristic pox and fungal lesions. **a** Grossly well circumscribed, popular, crusting pox lesions across the featherless skins (*white arrows*). **b** Feather skin demonstrating diffuse proliferation of the epidermis and follicular infundibula with keratinocytes containing eosinophilic intracytoplasmic inclusions (Bollinger bodies) and serocellular surface crust (*double head arrow*). **c** Inflammatory exudates associated with serocellular surface crust that contained abundant branching fungal hyphae and aggregations of bacteria

In the sample from *A. pacificus* (15–1526, and 15–1527), there were multifocal areas of inflammation and exudation associated with serocellular surface crust that contained abundant branching fungal hyphae and aggregations of bacteria (Fig. 1c). A PCR screening was conducted for the presence of fungal pathogen using the ITS region to amplify a segment of approximately 550 bp. Two samples (out of 6) were positive for fungal pathogens, and direct Sanger sequencing of the purified gel bands resulted in a 550 bp sequence after trimming off primer sequences (data not shown). These sequences were further verified using high-throughput NGS, and generated con tigs of 3,430 bp (15–1526; GenBank accession KX857213) and 5,188 bp (15–1527; GenBank accession KX857212). A BLASTn search for the bird coinfected with fungal pathogen (15–1526) returned multiple hits to various fungal species, all with very similar scores; however, the best match (88%) was to the *Phaeosphaeria nodorum* (GenBank Accession EU053989.1, and value ≤ e-153), a major necrotrophic fungal pathogen of wheat [21]. Similar search model for the fungal pathogen of bird 15–1527, demonstrated a highest hit (96%) to the *Metarhizium anisopliae* var. *anisopliae* (GenBank Accession AY884128.1, and value ≤ e-173), an entomopathogenic fungus [22].

### Identification of virus

Samples from six shearwater chicks of two different species, *A. carneipes* and *A. pacificus*, with evidence of gross well circumscribed, popular, crusting lesions across the feather skins (Fig. 1a), were biopsied, with blood and liver samples also collected. Histological examinations of the skin demonstrated focal to diffuse full thickness necrosis of the epidermis and a thick serocellular surface crust. A marked heterophilic rich inflammatory cellular response and exudation was present alongside abundant macrophages and perifollicular fibroplasia. In some areas there was focal proliferation of the adjacent epidermis associated with ballooning degeneration of keratinocytes with eosinophilic intracytoplasmic inclusions (Fig. 1b). A PCR screening was conducted for the presence of poxvirus, circovirus and reticuloendotheliosis virus, which are likely to cause this type of skin lesions. Two birds (*A. pacificus* 15*–*1526 and *A. carneipes* 15–1528) were positive by PCR targeting the 4b gene that encodes a core protein of ChPV, however, there were no evidence of either circovirus or reticuloendotheliosis for any of the samples used in this study. Direct Sanger sequencing of the purified gel bands resulted in a 578 bp sequence after trimming off primer sequences (data not shown). A BLASTn search with these sequences returned multiple hits to the 4b core gene from a variety of poxviruses, all with very similar scores; however, the best match was to the *Canarypox virus* 4b core protein gene ((bird 15–1526; similarity with AY318871 was 99% and identity score ≤ e-162), and bird 15–1528; similarity with LK021654 was 99% and identity score ≤ e-157)).

### Genome sequence and annotation of viruses

The *Shearwaterpox virus* complete genomes were assembled using CLC Genomics workbench 9.5.2 under La Trobe University Genomics Platform. The assembled complete genomes of SWPV-1 and −2 were 326,929 and 351,108 nt, respectively. The SWPV-1 and −2 complete genomes were annotated as described in the methods using CNPV as a reference genome (Additional file 1: Table S1 and Additional file 2: Table S2). We took a conservative approach to the annotation in order to minimize the inclusion of ORFs that were unlikely to represent functional genes. Table 1 lists the 310 and 312 genes annotated for SWPV-1 and −2, respectively. For the most part, these two new complete genomes are collinear to CNPV although there are a number of rearrangements of blocks of 1–6 genes in addition to insertions and deletions with respect to CNPV (Table 1). Comparison of the predicted proteins of SWPV-2 to orthologs in CNPV reveal the vast majority are >98% identical (aa), with more than 80 being completely conserved. In contrast, the orthologs of SWPV-1 only have an average aa identity of 67% to CNPV. However, with the lower average identity, greater genetic distance, comes a much greater range of variation in the level of identity and a significant number of predicted proteins are 80 – 90% identical (aa) to CNPV orthologs.

**Table 1:** Shearwaterpox virus (SWPV) genome annotations and comparative analysis of ORFs relative to CNPV genomes

SWPV1 synteny	SWPV2 synteny	CNPV synteny	CNPV BLAST hits	SWPV1 % identity	SWPV2 % identity	SWPV1 AA size	SWPV2 AA size	Reference AA size	notes
		CNPV001	CNPV001 hypothetical protein					72	
	SWPV2-001	CNPV002	CNPV002 hypothetical protein		92.941		171	171	
SWPV1-001	SWPV2-002	CNPV003	CNPV003 C-type lectin-like protein	32.044	85.99	181	208	204	
SWPV1-002		CNPV004	CNPV004 ankyrin repeat protein	56.458		468		514	
SWPV1-003	SWPV2-003	CNPV005	CNPV005 conserved hypothetical protein	87.387	99.55	220	222	222	
	SWPV2-004	CNPV006	CNPV006 hypothetical protein		88.71		134	182	SWPV2: C-terminus fragment, not likely translated
		CNPV007	CNPV007 ankyrin repeat protein					674	
SWPV1-004	SWPV2-005	CNPV008	CNPV008 C-type lectin-like protein	50	98.225	174	169	169	
	SWPV2-006	CNPV009	CNPV009 ankyrin repeat protein		99.564		688	688	
		CNPV010	CNPV010 ankyrin repeat protein					734	
	SWPV2-007	CNPV011	CNPV011 ankyrin repeat protein		99.147		586	586	
	SWPV2-008	CNPV012	CNPV012 hypothetical protein		100		189	189	
	SWPV2-009	CNPV013	CNPV013 hypothetical protein		98.81		168	168	
	SWPV2-010	CNPV014	CNPV014 immunoglobulin-like domain protein		99.184		490	490	
	SWPV2-011	CNPV015	CNPV015 ankyrin repeat protein		97.538		528	528	
SWPV1-005			CNPV035 C-type lectin-like protein	35.556		138		134	
SWPV1-006			CNPV318 ankyrin repeat protein	58.932		487		514	
SWPV1-007	SWPV2-012	CNPV016	CNPV016 C-type lectin-like protein	52.128	98.81	117	168	168	
SWPV1-008	SWPV2-013	CNPV017	CNPV017 ankyrin repeat protein	64.471	97.912	425	479	486	
SWPV1-009			CNPV295 ankyrin repeat protein	56.41		277		396	
	SWPV2-014	CNPV018	CNPV018 IL-10-like protein		90.805		190	191	
	SWPV2-015	CNPV019	CNPV019 ankyrin repeat protein		99.083		436	436	
SWPV1-010	SWPV2-016	CNPV020	CNPV020 ankyrin repeat protein	56.311	99.761	412	419	419	
SWPV1-011			CNPV320 Ig-like domain protein	31.656		483		469	
SWPV1-012	SWPV2-017	CNPV021	CNPV021 ankyrin repeat protein	62.313	99.626	528	535	535	
SWPV1-013	SWPV2-018	CNPV022	CNPV022 putative serpin	65.642	98.324	356	358	358	
SWPV1-014			PEPV260 ankyrin repeat protein	53.158		190		192	
SWPV1-015			CNPV011 ankyrin repeat protein	34		530		586	
	SWPV2-019	CNPV023	CNPV023 vaccinia C4L/C10L-like protein		98.595		424	427	
	SWPV2-020	CNPV024	CNPV024 hypothetical protein		96.629		178	178	
SWPV1-016	SWPV2-021	CNPV025	CNPV025 alpha-SNAP-like protein	57.491	98.667	304	300	300	
SWPV1-017	SWPV2-022	CNPV026	CNPV026 ankyrin repeat protein	54.271	98.953	397	382	382	
SWPV1-018	SWPV2-023	CNPV027	CNPV027 ankyrin repeat protein	59.375	98.722	646	626	626	
SWPV1-019	SWPV2-024	CNPV028	CNPV028 ankyrin repeat protein	57.618	99.164	408	365	362	
SWPV1-020	SWPV2-025	CNPV029	CNPV029 C-type lectin-like protein	50.35	99.296	142	142	142	
SWPV1-021	SWPV2-026	CNPV030	CNPV030 ankyrin repeat protein	63.72	98.529	345	340	340	
SWPV1-022	SWPV2-027	CNPV031	CNPV031 hypothetical protein	60.331	97.479	120	119	119	
SWPV1-023			CNPV013 conserved hypothetical protein	44.048		168		168	
SWPV1-024	SWPV2-028	CNPV032	CNPV032 Ig-like domain putative IFN-gamma binding protein	51.837	92.149	242	242	242	
SWPV1-025	SWPV2-029	CNPV033	CNPV033 Ig-like domain protein	48.095	93.496	238	246	246	
	SWPV2-030	CNPV034	CNPV034 ankyrin repeat protein		99.848		659	659	
	SWPV2-031	CNPV035	CNPV035 C-type lectin-like protein		94.776		133	134	
SWPV1-026	SWPV2-032	CNPV036	CNPV036 conserved hypothetical protein	48.235	98.947	88	95	95	
SWPV1-027	SWPV2-033	CNPV037	CNPV037 conserved hypothetical protein	63.068	99.441	178	179	179	
SWPV1-028	SWPV2-034	CNPV038	CNPV038 vaccinia C4L/C10L-like protein	54.523	99.516	411	413	413	
SWPV1-029	SWPV2-035	CNPV039	CNPV039 G protein-coupled receptor-like protein	67.284	97.859	323	327	327	
SWPV1-030	SWPV2-036	CNPV040	CNPV040 ankyrin repeat protein	57.36	93.401	589	591	591	SWPV2: High SNP Density
SWPV1-031	SWPV2-037	CNPV041	CNPV041 ankyrin repeat protein	66.284	98.605	432	430	430	
SWPV1-032	SWPV2-038	CNPV042	CNPV042 ankyrin repeat protein	72.712	99.339	608	605	605	
SWPV1-033	SWPV2-039	CNPV043	CNPV043 conserved hypothetical protein	74.627	99.005	202	201	201	
SWPV1-034	SWPV2-040	CNPV044	CNPV044 ankyrin repeat protein	67.316	99.583	470	480	480	
SWPV1-035	SWPV2-041	CNPV045	CNPV045 G protein-coupled receptor-like protein	65.231	100	331	332	332	
SWPV1-036	SWPV2-042	CNPV046	CNPV046 ankyrin repeat protein	68.08	98.667	452	450	450	
SWPV1-037	SWPV2-043	CNPV047	CNPV047 conserved hypothetical protein	65.6	99.194	125	124	124	
SWPV1-038	SWPV2-044	CNPV048	CNPV048 alkaline phosphodiesterase-like protein	68.238	98.502	804	801	801	
SWPV1-039	SWPV2-045	CNPV049	CNPV049 hypothetical protein	72.667	100	148	150	150	
SWPV1-040	SWPV2-046	CNPV050	CNPV050 ankyrin repeat protein	67.422	98.864	352	352	352	
SWPV1-041	SWPV2-047	CNPV051	CNPV051 DNase II-like protein	63.683	96.75	398	408	401	
SWPV1-042	SWPV2-048	CNPV052	CNPV052 C-type lectin-like protein	50	100	182	171	171	
SWPV1-043			FWPV ankyrin repeat protein	45		329		406	
SWPV1-044	SWPV2-049	CNPV053	CNPV053 conserved hypothetical protein	68.148	100	135	146	146	
SWPV1-045	SWPV2-050	CNPV054	CNPV054 conserved hypothetical protein	62.59	99.286	141	140	140	
SWPV1-046	SWPV2-051	CNPV055	CNPV055 conserved hypothetical protein	74.534	100	162	163	163	
SWPV1-047	SWPV2-052	CNPV056	CNPV056 dUTPase	80.986	98.621	155	145	145	
SWPV1-048	SWPV2-053	CNPV057	CNPV057 putative serpin	63.107	99.02	301	306	306	
SWPV1-049	SWPV2-054	CNPV058	CNPV058 bcl-2 like protein	51.744	98.857	174	180	175	
SWPV1-050	SWPV2-055	CNPV059	CNPV059 putative serpin	71.302	99.704	338	338	338	
SWPV1-051	SWPV2-056	CNPV060	CNPV060 conserved hypothetical protein	46.939	95.098	236	206	316	SWPV2: Large internal deletion, Translated but not likely functional
SWPV1-052	SWPV2-057	CNPV061	CNPV061 DNA ligase	80.995	98.761	567	565	565	
SWPV1-053	SWPV2-058	CNPV062	CNPV062 putative serpin	70.94	100	349	350	350	
SWPV1-054	SWPV2-059	CNPV063	CNPV063 hydroxysteroid dehydrogenase-like protein	71.348	99.441	359	358	358	
SWPV1-055	SWPV2-060	CNPV064	CNPV064 TGF-beta-like protein	56.897	98.587	272	283	282	
SWPV1-056	SWPV2-061	CNPV065	CNPV065 semaphorin-like protein	69.735	99.485	573	583	583	
SWPV1-057	SWPV2-062	CNPV066	CNPV066 hypothetical protein	37.349	98.519	139	399	405	SWPV1: Low BLAST hits, possible unique ORF
	SWPV2-063	CNPV067	CNPV067 hypothetical protein		100		57	57	
SWPV1-058			no significant BLAST hits					239	SWPV1: Possible Unique ORF
SWPV1-059	SWPV2-064	CNPV068	CNPV068 GNS1/SUR4-like protein	84.825	99.611	257	257	257	
SWPV1-060	SWPV2-065	CNPV069	CNPV069 late transcription factor VLTF-2	87.5	100	154	155	155	
SWPV1-061	SWPV2-066	CNPV070	CNPV070 putative rifampicin resistance protein, IMV assembly	88.065	100	553	551	551	
SWPV1-062	SWPV2-067	CNPV071	CNPV071 mRNA capping enzyme small subunit	89.273	100	289	289	289	
	SWPV2-068	CNPV072	CNPV072 CC chemokine-like protein		96.262		132	312	SWPV2: N-terminus fragment
SWPV1-063	SWPV2-069	CNPV073	CNPV073 hypothetical protein	45.263	100	110	109	109	
SWPV1-064	SWPV2-070	CNPV074	CNPV074 NPH-I, transcription termination factor	92.756	99.685	635	635	635	
SWPV1-065	SWPV2-071	CNPV075	CNPV075 mutT motif putative gene expression regulator	79.295	100	226	228	230	
SWPV1-066	SWPV2-072	CNPV076	CNPV076 mutT motif	84.549	99.569	233	232	232	
		CNPV077	CNPV077 hypothetical protein					78	
SWPV1-067			CNPV011 ankyrin repeat protein	29.806		435		586	
SWPV1-068	SWPV2-073	CNPV078	CNPV078 RNA polymerase subunit RPO18	82.39	100	161	160	160	
	SWPV2-074	CNPV079	CNPV079 Ig-like domain protein		94.161		274	272	
SWPV1-069	SWPV2-075	CNPV080	CNPV080 early transcription factor small subunit VETFS	96.682	100	633	633	633	
	SWPV2-076	CNPV081	CNPV081 Ig-like domain protein		97.006		334	333	
SWPV1-070	SWPV2-077	CNPV082	CNPV082 NTPase, DNA replication	88.818	99.748	790	794	794	
SWPV1-071	SWPV2-078	CNPV083	CNPV083 CC chemokine-like protein	60.352	91.855	223	221	221	
SWPV1-072			CNPV215 CC chemokine-like protein	30.994		195		204	
SWPV1-073	SWPV2-079	CNPV084	CNPV084 uracil DNA glycosylase	86.364	97.706	220	218	218	
SWPV1-074	SWPV2-080	CNPV085	CNPV085 putative RNA phosphatase	67.895	74.312	245	303	403	SWPV2: High SNP Density
SWPV1-075			CNPV216 conserved hypothetical protein	39.225		398		404	
SWPV1-076	SWPV2-081	CNPV086	CNPV086 TNFR-like protein	67.327	71.569	103	112	117	
	SWPV2-082	CNPV087	CNPV087 putative glutathione peroxidase		98.473		131	198	SWPV2: C-terminus fragment, not likely translated
SWPV1-077			CNPV227 N1R/p28-like protein	74.638		256		359	
SWPV1-078	SWPV2-083	CNPV088	CNPV088 conserved hypothetical protein	55.769	97	104	100	100	
SWPV1-079	SWPV2-084	CNPV089	CNPV089 conserved hypothetical protein	64.935	100	164	159	159	
SWPV1-080	SWPV2-085	CNPV090	CNPV090 conserved hypothetical protein	62.393	100	124	127	127	
SWPV1-081	SWPV2-086	CNPV091	CNPV091 HT motif protein	64.634	100	77	83	83	
SWPV1-082	SWPV2-087	CNPV092	CNPV092 conserved hypothetical protein	64.901	97.945	140	146	146	
SWPV1-083	SWPV2-088	CNPV093	CNPV093 virion protein	60.37	99.625	270	267	267	
SWPV1-084	SWPV2-089	CNPV094	CNPV094 T10-like protein	75	98.909	282	275	275	
SWPV1-085	SWPV2-090	CNPV095	CNPV095 conserved hypothetical protein	71.111	100	47	45	45	
SWPV1-086	SWPV2-091	CNPV096	CNPV096 ubiquitin	100	100	77	85	85	
SWPV1-087	SWPV2-092	CNPV097	CNPV097 conserved hypothetical protein	70.031	99.705	298	339	339	
SWPV1-088	SWPV2-093	CNPV098	CNPV098 hypothetical protein	67.442	98.75	61	80	80	
SWPV1-089	SWPV2-094	CNPV099	CNPV099 beta-NGF-like protein	62.162	97.949	186	195	195	
SWPV1-090	SWPV2-095	CNPV100	CNPV100 putative interleukin binding protein	51.176	98.225	211	168	169	
	SWPV2-096	CNPV101	CNPV101 hypothetical protein		98.824		85	85	
SWPV1-091	SWPV2-097	CNPV102	CNPV102 conserved hypothetical protein	54.167	99.048	102	105	105	
SWPV1-092	SWPV2-098	CNPV103	CNPV103 N1R/p28-like protein	62.304	98.947	188	190	190	
SWPV1-093	SWPV2-099	CNPV104	CNPV104 putative glutaredoxin 2, virion morphogenesis	86.4	99.2	125	125	125	
SWPV1-094	SWPV2-100	CNPV105	CNPV105 conserved hypothetical protein	77.35	98.718	234	234	234	
SWPV1-095	SWPV2-101	CNPV106	CNPV106 putative elongation factor	76.829	98.039	103	102	102	
	SWPV2-102	CNPV107	CNPV107 hypothetical protein		100		77	77	
SWPV1-096			PEPV083 transforming growth factor B	64		444		336	
SWPV1-097	SWPV2-103	CNPV108	CNPV108 putative metalloprotease, virion morphogenesis	85.489	100	633	632	632	
SWPV1-098	SWPV2-104	CNPV109	CNPV109 NPH-II, RNA helicase	86.05	99.706	681	681	681	
SWPV1-099	SWPV2-105	CNPV110	CNPV110 virion core proteinase	87.441	99.763	421	422	422	
SWPV1-100	SWPV2-106	CNPV111	CNPV111 DNA-binding protein	80.612	99.488	391	391	391	
SWPV1-101	SWPV2-107	CNPV112	CNPV112 putative IMV membrane protein	81.481	100	81	81	81	
SWPV1-102	SWPV2-108	CNPV113	CNPV113 thymidine kinase	75.978	99.441	181	179	179	
SWPV1-103	SWPV2-109	CNPV114	CNPV114 HT motif protein	69.62	100	79	82	82	
SWPV1-104	SWPV2-110	CNPV115	CNPV115 DNA-binding phosphoprotein	71.429	82.353	282	289	289	SWPV2: High SNP density
SWPV1-105	SWPV2-111	CNPV116	CNPV116 unnamed protein product	73.913	98.551	66	69	69	
SWPV1-106	SWPV2-112	CNPV117	CNPV117 DNA-binding virion protein	88.854	99.677	314	310	310	
SWPV1-107	SWPV2-113	CNPV118	CNPV118 conserved hypothetical protein	75.762	99.387	656	652	653	
SWPV1-108	SWPV2-114	CNPV119	CNPV119 virion core protein	83.969	100	131	131	131	
SWPV1-109	SWPV2-115	CNPV120	CNPV120 putative IMV redox protein, virus assembly	80.851	100	94	93	93	
SWPV1-110	SWPV2-116	CNPV121	CNPV121 DNA polymerase	89.17	99.899	988	988	988	
SWPV1-111		CNPV122	CNPV122 putative membrane protein	83.088		273		274	
SWPV1-112	SWPV2-117	CNPV123	CNPV123 conserved hypothetical protein	82.312	85.336	571	502	571	SWPV2: High SNP density
SWPV1-113	SWPV2-118	CNPV124	CNPV124 variola B22R-like protein	67	98.957	1906	1916	1918	
SWPV1-114	SWPV2-119	CNPV125	CNPV125 variola B22R-like protein	71.669	99.66	1742	1767	1767	
SWPV1-115	SWPV2-120	CNPV126	CNPV126 variola B22R-like protein	64.456	98.847	1902	1839	1951	SWPV2: N-terminus fragment
	SWPV2-121		CNPV126 variola B22R-like protein		96		153	1951	SWPV2: C-terminus fragment, not likely translated
SWPV1-116	SWPV2-122	CNPV127	CNPV127 RNA polymerase subunit RPO30	96.154	100	182	182	182	
SWPV1-117	SWPV2-123	CNPV128	CNPV128 conserved hypothetical protein	77.072	98.752	742	721	721	SWPV2: High SNP Density
SWPV1-118	SWPV2-124	CNPV129	CNPV129 poly(A) polymerase large subunit PAPL	83.898	99.788	472	472	472	
SWPV1-119	SWPV2-125	CNPV130	CNPV130 DNA-binding virion core protein	76.471	100	114	119	119	
SWPV1-120	SWPV2-126	CNPV131	CNPV131 conserved hypothetical protein	64.115	99.517	212	207	207	
SWPV1-121	SWPV2-127	CNPV132	CNPV132 conserved hypothetical protein	81.081	99.324	151	148	148	
SWPV1-122	SWPV2-128	CNPV133	CNPV133 conserved hypothetical protein	73.737	100	90	99	99	
SWPV1-123	SWPV2-129	CNPV134	CNPV134 variola B22R-like protein	65.517	99.001	1774	1801	1801	
SWPV1-124	SWPV2-130	CNPV135	CNPV135 putative palmitylated EEV envelope lipase	89.418	99.735	378	378	378	
SWPV1-125	SWPV2-131	CNPV136	CNPV136 putative EEV maturation protein	75.602	99.68	622	625	625	
SWPV1-126	SWPV2-132	CNPV137	CNPV137 conserved hypothetical protein	62.26	98.925	467	462	465	
SWPV1-127	SWPV2-133	CNPV138	CNPV138 putative serine/threonine protein kinase, virus assembly	83.632	100	445	444	444	
SWPV1-128	SWPV2-134	CNPV139	CNPV139 conserved hypothetical protein	81.69	100	213	213	213	
SWPV1-129	SWPV2-135	CNPV140	CNPV140 conserved hypothetical protein	78.788	100	65	66	66	
SWPV1-130	SWPV2-136	CNPV141	CNPV141 HAL3-like domain protein	88.333	100	182	184	184	
SWPV1-131			no significant BLAST hits	28		101		571	SWPV1: Possible Unique ORF
SWPV1-132	SWPV2-137	CNPV142	CNPV142 N1R/p28-like protein	48.266	98.442	314	321	321	
SWPV1-133	SWPV2-138	CNPV143	CNPV143 ankyrin repeat protein	54.103	98.361	634	671	671	
SWPV1-134	SWPV2-139	CNPV144	CNPV144 ankyrin repeat protein	59.011	99.281	562	556	556	
SWPV1-135	SWPV2-140	CNPV145	CNPV145 conserved hypothetical protein	75.814	100	439	440	440	
SWPV1-136	SWPV2-141	CNPV146	CNPV146 RNA polymerase subunit RPO7	88.525	100	66	62	62	
SWPV1-137	SWPV2-142	CNPV147	CNPV147 conserved hypothetical protein	80.851	100	188	188	188	
SWPV1-138	SWPV2-143	CNPV148	CNPV148 virion core protein	86.533	100	347	348	348	
	SWPV2-144	CNPV149	CNPV149 putative thioredoxin binding protein		99.673		306	306	
		CNPV150	CNPV150 ankyrin repeat protein					351	
	SWPV2-145	CNPV151	CNPV151 ankyrin repeat protein		99.029		412	412	
	SWPV2-146	CNPV152	CNPV152 hypothetical protein		98		149	187	SWPV2: C-terminus fragment, not likely translated
	SWPV2-147	CNPV153	CNPV153 Rep-like protein		99.359		312	312	
SWPV1-139			CNPV159 N1R/p28-like protein	78.488		333		337	
SWPV1-140			FWPV121 CC chemokine-like protein	46		93		121	
SWPV1-141	SWPV2-148	CNPV154	CNPV154 variola B22R-like protein	90.067	98.286	1939	875	1928	SWPV2: N-terminus fragment/SWPV1: Low SNP Density
SWPV1-142	SWPV2-149	CNPV155	CNPV155 variola B22R-like protein	82.427	99.454	1810	1831	1830	
	SWPV2-150	CNPV156	CNPV156 hypothetical protein		96.287		834	832	
	SWPV2-151	CNPV157	CNPV157 TGF-beta-like protein		87.679		343	349	
		CNPV158	CNPV158 TGF-beta-like protein					172	
		CNPV159	CNPV159 N1R/p28-like protein					337	
		CNPV160	CNPV160 N1R/p28-like protein					396	
	SWPV2-152	CNPV161	CNPV161 TGF-beta-like protein		99.441		358	358	
	SWPV2-153	CNPV162	CNPV162 TGF-beta-like protein		97.987		149	149	
		CNPV163	CNPV163 hypothetical protein					92	
		CNPV164	CNPV164 hypothetical protein					98	
	SWPV2-154	CNPV165	CNPV165 N1R/p28-like protein		98.75		320	346	SWPV2: C-terminus fragment, not likely translated
SWPV1-143	SWPV2-155	CNPV166	CNPV166 Ig-like domain protein	96.812	95.652	345	345	345	SWPV1: Low SNP Density
SWPV1-144	SWPV2-156	CNPV167	CNPV167 Ig-like domain protein	94.767	88.372	172	168	171	SWPV1: Low SNP Density
	SWPV2-157	CNPV168	CNPV168 N1R/p28-like protein		96		350	358	
SWPV1-145		CNPV169	CNPV169 N1R/p28-like protein	83.578		337		332	SWPV1: CNPV-168/169 Fusion
SWPV1-146	SWPV2-158	CNPV170	CNPV170 thymidylate kinase	100	100	121	212	212	SWPV1: N-terminus fragment
SWPV1-147	SWPV2-159	CNPV171	CNPV171 late transcription factor VLTF-1	96.923	100	260	260	260	
SWPV1-148	SWPV2-160	CNPV172	CNPV172 putative myristylated protein	83.125	99.403	336	335	335	
SWPV1-149	SWPV2-161	CNPV173	CNPV173 putative myristylated IMV envelope protein	91.358	98.354	243	243	243	
SWPV1-150	SWPV2-162	CNPV174	CNPV174 conserved hypothetical protein	47.917	100	96	96	96	
SWPV1-151	SWPV2-163	CNPV175	CNPV175 conserved hypothetical protein	84.158	100	303	303	303	
SWPV1-152	SWPV2-164	CNPV176	CNPV176 DNA-binding virion core protein	87.747	100	253	252	252	
SWPV1-153	SWPV2-165	CNPV177	CNPV177 conserved hypothetical protein	84.733	100	131	130	130	
SWPV1-154	SWPV2-166	CNPV178	CNPV178 putative IMV membrane protein	85.135	100	148	148	148	
SWPV1-155	SWPV2-167	CNPV179	CNPV179 poly(A) polymerase small subunit PAPS	88.667	100	300	302	302	
SWPV1-156	SWPV2-168	CNPV180	CNPV180 RNA polymerase subunit RPO22	87.634	99.462	186	186	186	
SWPV1-157	SWPV2-169	CNPV181	CNPV181 conserved hypothetical protein	82.353	100	136	136	136	
SWPV1-158	SWPV2-170	CNPV182	CNPV182 RNA polymerase subunit RPO147	93.866	99.922	1288	1288	1288	
SWPV1-159	SWPV2-171	CNPV183	CNPV183 putative protein-tyrosine phosphatase, virus assembly	85.542	100	166	166	166	
SWPV1-160	SWPV2-172	CNPV184	CNPV184 conserved hypothetical protein	91.534	100	190	189	189	
SWPV1-161	SWPV2-173	CNPV185	CNPV185 ankyrin repeat protein	32.632	96.341	337	328	328	
SWPV1-162	SWPV2-174	CNPV186	CNPV186 IMV envelope protein	100	100	329	330	330	
SWPV1-163	SWPV2-175	CNPV187	CNPV187 RNA polymerase associated protein RAP94	91.114	99.75	799	799	799	
SWPV1-164	SWPV2-176	CNPV188	CNPV188 late transcription factor VLTF-4	70.115	92.941	170	170	170	
SWPV1-165	SWPV2-177	CNPV189	CNPV189 DNA topoisomerase	88.608	99.684	316	316	316	
SWPV1-166	SWPV2-178	CNPV190	CNPV190 conserved hypothetical protein	77.124	99.346	153	153	153	
SWPV1-167	SWPV2-179	CNPV191	CNPV191 conserved hypothetical protein	70.874	99.029	103	103	103	
SWPV1-168	SWPV2-180	CNPV192	CNPV192 mRNA capping enzyme large subunit	88.221	99.764	848	846	846	
SWPV1-169	SWPV2-181	CNPV193	CNPV193 HT motif protein	72.619	100	104	106	106	
SWPV1-170	SWPV2-182	CNPV194	CNPV194 virion protein	71.223	100	139	140	140	
SWPV1-171	SWPV2-183	CNPV195	CNPV195 hypothetical protein	51.2	98.611	139	144	144	
SWPV1-172	SWPV2-184	CNPV196	CNPV196 conserved hypothetical protein	62.963	100	189	190	190	
SWPV1-173	SWPV2-185	CNPV197	CNPV197 N1R/p28-like protein	61.679	97.818	279	275	275	
SWPV1-174	SWPV2-186	CNPV198	CNPV198 C-type lectin-like protein	55.844	99.359	159	156	156	
SWPV1-175	SWPV2-187	CNPV199	CNPV199 deoxycytidine kinase-like protein	79.111	100	222	225	225	
SWPV1-176	SWPV2-188	CNPV200	CNPV200 Rep-like protein	72.903	97.59	152	166	166	
SWPV1-177	SWPV2-189	CNPV201	CNPV201 conserved hypothetical protein	60	97.661	197	167	192	
SWPV1-178	SWPV2-190	CNPV202	CNPV202 N1R/p28-like protein	69.203	99.638	275	276	276	
SWPV1-179	SWPV2-191	CNPV203	CNPV203 N1R/p28-like protein	64.935	99.738	380	382	382	
SWPV1-180	SWPV2-192	CNPV204	CNPV204 conserved hypothetical protein	53.226	100	53	61	61	
SWPV1-181	SWPV2-193	CNPV205	CNPV205 N1R/p28-like protein	71.885	99.371	317	318	318	
SWPV1-182	SWPV2-194	CNPV206	CNPV206 putative photolyase	84.989	99.364	464	472	472	
SWPV1-183			CNPV081 Ig-like domain protein	53.988		332		333	
SWPV1-184	SWPV2-195	CNPV207	CNPV207 N1R/p28-like protein	64.535	98.235	193	173	183	
SWPV1-185	SWPV2-196	CNPV208	CNPV208 conserved hypothetical protein	52.239	97.5	172	200	200	
SWPV1-186	SWPV2-197	CNPV209	CNPV209 N1R/p28-like protein	65.686	100	311	310	310	
SWPV1-187	SWPV2-198	CNPV210	CNPV210 N1R/p28-like protein	74.419	99.237	130	131	131	
SWPV1-188	SWPV2-199	CNPV211	CNPV211 conserved hypothetical protein	49.02	98.148	54	54	54	
SWPV1-189	SWPV2-200	CNPV212	CNPV212 N1R/p28-like protein	76.136	98.295	175	176	176	
SWPV1-190			no significant BLAST hits			70			SWPV1: Possible Unique ORF
SWPV1-191	SWPV2-201	CNPV213	CNPV213 deoxycytidine kinase-like protein	58.768	99.539	216	216	217	
	SWPV2-202	CNPV214	CNPV214 vaccinia C4L/C10L-like protein		99.438		356	356	
SWPV1-192			CNPV012 conserved hypothetical protein	37.41		165		189	
SWPV1-193			CNPV223 ankyrin repeat protein	31.579		674		847	
SWPV1-194	SWPV2-203	CNPV215	CNPV215 CC chemokine-like protein	49.751	96.078	202	204	204	
	SWPV2-204	CNPV216	CNPV216 conserved hypothetical protein		98.762		401	404	
	SWPV2-205	CNPV217	CNPV217 N1R/p28-like protein		95.152		330	330	
SWPV1-195			CNPV223 ankyrin repeat protein	38.474		729		847	SWPV1: N-terminus fragment
SWPV1-196	SWPV2-206	CNPV218	CNPV218 N1R/p28-like protein	66.667	99.522	318	223	437	SWPV2: N-terminus fragment
SWPV1-197			CNPV228 N1R/p28-like protein	53		161		371	SWPV1: N-terminus fragment
SWPV1-198			CNPV160 N1R/p28-like protein	79.293		367		396	SWPV1: Fragment/CNPV-220/221 Fusion
SWPV1-199			CNPV160 N1R/p28-like protein	66.582		360		396	SWPV1: Paralog to SWPV1-198?
SWPV1-200			CNPV161 TGF-beta-like protein	36.882		256		358	
SWPV1-201			CNPV162 TGF-beta-like protein	50		141		149	
SWPV1-202			no significant BLAST hits			98			SWPV1: Possible Unique ORF
	SWPV2-207	CNPV219	CNPV219 N1R/p28-like protein		99.713		349	349	
	SWPV2-208	CNPV220	CNPV220 N1R/p28-like protein		80.263		85	178	SWPV2: N-terminus fragment
	SWPV2-209	CNPV221	CNPV221 N1R/p28-like protein		94.231		213	281	SWPV2: N-terminus fragment
	SWPV2-210	CNPV222	CNPV222 N1R/p28-like protein		99.649		285	285	
	SWPV2-211	CNPV223	CNPV223 ankyrin repeat protein		98.819		847	847	
SWPV1-203	SWPV2-212	CNPV224	CNPV224 hypothetical protein	50.382	100	126	239	239	
	SWPV2-213	CNPV225	CNPV225 N1R/p28-like protein		74.038		94	159	SWPV2: N-terminus fragment
	SWPV2-214	CNPV226	CNPV226 N1R/p28-like protein		96.825		126	134	
		CNPV227	CNPV227 N1R/p28-like protein					359	
		CNPV228	CNPV228 N1R/p28-like protein					371	
SWPV1-204	SWPV2-215	CNPV229	CNPV229 ankyrin repeat protein	44.498	97.926	423	434	434	
	SWPV2-216	CNPV230	CNPV230 hypothetical protein		98.462		65	65	
SWPV1-205	SWPV2-217	CNPV231	CNPV231 MyD116-like domain protein	72.222	98.101	100	158	158	SWPV1: large in-frame deletions
SWPV1-206	SWPV2-218	CNPV232	CNPV232 CC chemokine-like protein	59.024	93.137	205	204	204	
SWPV1-207	SWPV2-219	CNPV233	CNPV233 ankyrin repeat protein	56.936	99.788	476	471	471	
	SWPV2-220	CNPV234	CNPV234 ankyrin repeat protein		100		508	508	SWPV2: High SNP Density
SWPV1-208			PEPV008 vaccinia C4L/C10L-like protein	55		420		411	
	SWPV2-221	CNPV235	CNPV235 conserved hypothetical protein		88.426		432	432	
SWPV1-209	SWPV2-222	CNPV236	CNPV236 ribonucleotide reductase small subunit	83.282	95.666	324	323	323	
	SWPV2-223	CNPV237	CNPV237 ankyrin repeat protein		97.732		441	441	
SWPV1-210			CNPV234 ankyrin repeat protein	30.545		559		508	
SWPV1-211	SWPV2-224	CNPV238	CNPV238 late transcription factor VLTF-3	95.111	100	225	225	225	
SWPV1-212	SWPV2-225	CNPV239	CNPV239 virion redox protein	80.282	100	72	75	75	
SWPV1-213	SWPV2-226	CNPV240	CNPV240 virion core protein P4b	88.788	99.848	660	659	659	
SWPV1-214	SWPV2-227	CNPV241	CNPV241 immunodominant virion protein	47.368	99.07	242	215	215	
SWPV1-215	SWPV2-228	CNPV242	CNPV242 RNA polymerase subunit RPO19	88.166	98.817	169	169	169	
SWPV1-216	SWPV2-229	CNPV243	CNPV243 conserved hypothetical protein	81.501	98.928	373	373	373	
SWPV1-217	SWPV2-230	CNPV244	CNPV244 early transcription factor large subunit VETFL	95.91	100	709	709	709	
SWPV1-218	SWPV2-231	CNPV245	CNPV245 intermediate transcription factor VITF-3	90.667	99.667	300	300	300	
SWPV1-219	SWPV2-232	CNPV246	CNPV246 putative IMV membrane protein	80	98.667	76	75	75	
SWPV1-220	SWPV2-233	CNPV247	CNPV247 virion core protein P4a	81.494	99.664	897	893	893	
SWPV1-221	SWPV2-234	CNPV248	CNPV248 conserved hypothetical protein	78.723	100	281	279	279	
SWPV1-222	SWPV2-235	CNPV249	CNPV249 virion protein	74.269	99.405	167	168	168	
SWPV1-223	SWPV2-236	CNPV250	CNPV250 conserved hypothetical protein	36.082	94.595	73	56	99	SWPV2: N-terminus fragment
SWPV1-224	SWPV2-237	CNPV251	CNPV251 putative IMV membrane protein	69.565	100	69	69	69	
SWPV1-225	SWPV2-238	CNPV252	CNPV252 putative IMV membrane protein	68.478	98.913	92	92	92	
SWPV1-226	SWPV2-239	CNPV253	CNPV253 putative IMV membrane virulence factor	73.585	98.113	53	53	53	
SWPV1-227	SWPV2-240	CNPV254	CNPV254 conserved hypothetical protein	75	98.958	96	96	96	
SWPV1-228	SWPV2-241	CNPV255	CNPV255 predicted myristylated protein	84.282	99.728	368	368	368	
SWPV1-229	SWPV2-242	CNPV256	CNPV256 putative phosphorylated IMV membrane protein	81.006	100	188	192	192	
SWPV1-230	SWPV2-243	CNPV257	CNPV257 DNA helicase, transcriptional elongation	87.229	99.784	462	462	462	
SWPV1-231	SWPV2-244	CNPV258	CNPV258 conserved hypothetical protein	77.647	100	86	89	89	
SWPV1-232	SWPV2-245	CNPV259	CNPV259 DNA polymerase processivity factor	81.86	100	432	112	434	
SWPV1-233	SWPV2-246	CNPV260	CNPV260 conserved hypothetical protein	91.071	99.77	112	434	112	
SWPV1-234	SWPV2-247	CNPV261	CNPV261 Holliday junction resolvase protein	80.405	100	151	152	152	
SWPV1-235	SWPV2-248	CNPV262	CNPV262 intermediate transcription factor VITF-3	86.126	100	383	383	383	
SWPV1-236	SWPV2-249	CNPV263	CNPV263 RNA polymerase subunit RPO132	94.301	100	1158	1157	1157	
SWPV1-237	SWPV2-250	CNPV264	CNPV264 A type inclusion-like protein	81.015	99.502	602	601	603	
SWPV1-238	SWPV2-251	CNPV265	CNPV265 A type inclusion-like/fusion protein	67.015	99.789	471	475	475	
SWPV1-239	SWPV2-252	CNPV266	CNPV266 conserved hypothetical protein	89.286	99.286	140	140	140	
SWPV1-240	SWPV2-253	CNPV267	CNPV267 RNA polymerase subunit RPO35	77.558	99.016	303	305	305	
SWPV1-241	SWPV2-254	CNPV268	CNPV268 conserved hypothetical protein	73.529	100	72	75	75	
SWPV1-242	SWPV2-255	CNPV269	CNPV269 conserved hypothetical protein	70.796	100	113	113	113	
SWPV1-243	SWPV2-256	CNPV270	CNPV270 conserved hypothetical protein	70.588	100	119	120	120	
SWPV1-244	SWPV2-257	CNPV271	CNPV271 DNA packaging protein	89.963	99.648	272	284	284	
SWPV1-245	SWPV2-258	CNPV272	CNPV272 C-type lectin-like EEV protein	76.136	99.448	182	181	181	
SWPV1-246			CNPV012 conserved hypothetical protein	30.147		172		189	
SWPV1-247	SWPV2-259	CNPV273	CNPV273 conserved hypothetical protein	62.816	99.635	276	274	274	
SWPV1-248	SWPV2-260	CNPV274	CNPV274 putative tyrosine protein kinase	63.197	99.628	286	269	269	
SWPV1-249	SWPV2-261	CNPV275	CNPV275 putative serpin	72.271	99.408	340	338	338	
SWPV1-250	SWPV2-262	CNPV276	CNPV276 conserved hypothetical protein	56.667	100	227	252	252	
SWPV1-251	SWPV2-263	CNPV277	CNPV277 G protein-coupled receptor-like protein	90	99.677	310	310	310	
SWPV1-252	SWPV2-264	CNPV278	CNPV278 conserved hypothetical protein	89.691	98.958	97	96	96	
SWPV1-253	SWPV2-265	CNPV279	CNPV279 beta-NGF-like protein	63.415	100	167	169	169	
SWPV1-254	SWPV2-266	CNPV280	CNPV280 HT motif protein	67.692	99.231	134	130	130	
SWPV1-255	SWPV2-267	CNPV281	CNPV281 conserved hypothetical protein	71.728	99.533	192	214	214	
SWPV1-256	SWPV2-268	CNPV282	CNPV282 HT motif protein	71.552	100	118	120	120	
SWPV1-257	SWPV2-269	CNPV283	CNPV283 CC chemokine-like protein	63.208	100	110	111	111	
SWPV1-258	SWPV2-270	CNPV284	CNPV284 putative interleukin binding protein	37.405	90.769	192	193	195	
SWPV1-259	SWPV2-271	CNPV285	CNPV285 EGF-like protein	62.992	99.206	123	126	126	
SWPV1-260	SWPV2-272	CNPV286	CNPV286 putative serine/threonine protein kinase	76.744	99.672	303	305	305	
SWPV1-261	SWPV2-273	CNPV287	CNPV287 conserved hypothetical protein	73.248	98.758	165	160	161	
SWPV1-262	SWPV2-274	CNPV288	CNPV288 C-type lectin-like protein	52.414	88.435	163	147	147	
SWPV1-263	SWPV2-275	CNPV289	CNPV289 putative interleukin binding protein	58.993	99.281	132	139	139	
SWPV1-264	SWPV2-276	CNPV290	CNPV290 conserved hypothetical protein	84	83.784	75	75	75	
SWPV1-265	SWPV2-277	CNPV291	CNPV291 ankyrin repeat protein	48.067	98.99	613	594	594	
SWPV1-266	SWPV2-278	CNPV292	CNPV292 hypothetical protein	37.209	100	101	74	74	
SWPV1-267	SWPV2-279	CNPV293	CNPV293 ankyrin repeat protein	55.634	99.648	305	284	284	
SWPV1-268	SWPV2-280	CNPV294	CNPV294 ankyrin repeat protein	68.447	99.07	424	430	430	
SWPV1-269			PIPV223 host range protein	51		138		143	
SWPV1-270			FWPV217 hypothetical protein	50		330		328	
SWPV1-271	SWPV2-281	CNPV295	CNPV295 ankyrin repeat protein	57.736	100	264	396	396	
SWPV1-272	SWPV2-282	CNPV296	CNPV296 ankyrin repeat protein	67.195	99.127	438	458	458	
SWPV1-273	SWPV2-283	CNPV297	CNPV297 ankyrin repeat protein	54.972	99.457	717	737	737	
SWPV1-274	SWPV2-284	CNPV298	CNPV298 ankyrin repeat protein	64.591	99.825	573	571	571	
SWPV1-275	SWPV2-285	CNPV299	CNPV299 putative serine/threonine protein kinase	67.893	99.333	303	300	300	
SWPV1-276	SWPV2-286	CNPV300	CNPV300 ankyrin repeat protein	75.82	98.77	253	244	244	
SWPV1-277			CNPV219 N1R/p28-like protein	28.467		142		349	
SWPV1-278			CNPV228 N1R/p28-like protein	43.038		87		371	
SWPV1-279			TKPV163 ankyrin repeat protein	40		432		434	
SWPV1-280	SWPV2-287	CNPV301	CNPV301 ankyrin repeat protein	59.546	99.241	510	527	527	
SWPV1-281	SWPV2-288	CNPV302	CNPV302 conserved hypothetical protein	45.026	100	175	193	193	
SWPV1-282	SWPV2-289	CNPV303	CNPV303 ankyrin repeat protein	68.938	99.4	499	500	500	
SWPV1-283	SWPV2-290	CNPV304	CNPV304 ankyrin repeat protein	62.105	99.785	476	466	466	
SWPV1-284	SWPV2-291	CNPV305	CNPV305 N1R/p28-like protein	54.545	100	261	262	262	
SWPV1-285	SWPV2-292	CNPV306	CNPV306 hypothetical protein	30.769	98.611	73	72	72	
SWPV1-286	SWPV2-293	CNPV307	CNPV307 C-type lectin-like protein	55.828	100	165	154	154	
SWPV1-287	SWPV2-294	CNPV308	CNPV308 ankyrin repeat protein	58.757	99.44	359	357	357	
SWPV1-288	SWPV2-295	CNPV309	CNPV309 ankyrin repeat protein	69.388	100	195	196	196	
SWPV1-289	SWPV2-296	CNPV310	CNPV310 ankyrin repeat protein	47.359	99.255	540	537	537	
SWPV1-290	SWPV2-297	CNPV311	CNPV311 EFc-like protein	54.4	99.194	125	124	124	
SWPV1-291	SWPV2-298	CNPV312	CNPV312 conserved hypothetical protein	53.704	98.795	168	166	166	
SWPV1-292	SWPV2-299	CNPV313	CNPV313 Ig-like domain protein	69.43	98.165	213	218	218	
SWPV1-293	SWPV2-300	CNPV314	CNPV314 ankyrin repeat protein	71.552	99.829	580	629	584	
SWPV1-294			CNPV011 ankyrin repeat protein	32		513		586	
SWPV1-295	SWPV2-301	CNPV315	CNPV315 G protein-coupled receptor-like protein	59.17	99.365	315	315	315	
SWPV1-296			CNPV014 Ig-like domain protein	59.624		230		490	
SWPV1-297			CNPV014 Ig-like domain protein	59.641		240		490	
SWPV1-298			CNPV015 ankyrin repeat protein	45.455		74		528	
SWPV1-299			CNPV150 ankyrin repeat protein	36.364		84		351	
SWPV1-300	SWPV2-302	CNPV316	CNPV316 ankyrin repeat protein	35.294	99.632	162	544	544	
	SWPV2-303	CNPV317	CNPV317 hypothetical protein		100		55	55	
	SWPV2-304	CNPV318	CNPV318 ankyrin repeat protein		98.054		514	514	
	SWPV2-305	CNPV319	CNPV319 ankyrin repeat protein		97.638		637	739	SWPV2: C-terminus fragment, not likely translated
SWPV1-301			PIPV253 EFc-like protein	69		124		124	
SWPV1-302			CNPV015 ankyrin repeat protein	45.276		520		528	
SWPV1-303			CNPV223 ankyrin repeat protein	40		480		847	
SWPV1-304	SWPV2-306	CNPV320	CNPV320 Ig-like domain protein	76.858	99.787	468	469	469	
	SWPV2-307	CNPV321	CNPV321 EFc-like protein		99.194		124	124	
	SWPV2-308	CNPV322	CNPV322 ankyrin repeat protein		98.408		689	690	
SWPV1-305			CNPV035 C-type lectin-like protein	35.556		138		134	
SWPV1-306			CNPV008 C-type lectin-like protein	50		174		169	
SWPV1-307	SWPV2-309	CNPV323	CNPV323 conserved hypothetical protein	75.61	93.651	84	186	182	
SWPV1-308	SWPV2-310	CNPV324	CNPV324 conserved hypothetical protein	87.387	99.55	220	222	222	
SWPV1-309		CNPV325	CNPV325 ankyrin repeat protein	56.458		468		514	
SWPV1-310	SWPV2-311	CNPV326	CNPV326 C-type lectin-like protein	32.044	85.99	181	208	204	
	SWPV2-312	CNPV327	CNPV327 hypothetical protein		92.941		171	171	
		CNPV328	CNPV328 hypothetical protein					72	

**Fig. 2:**
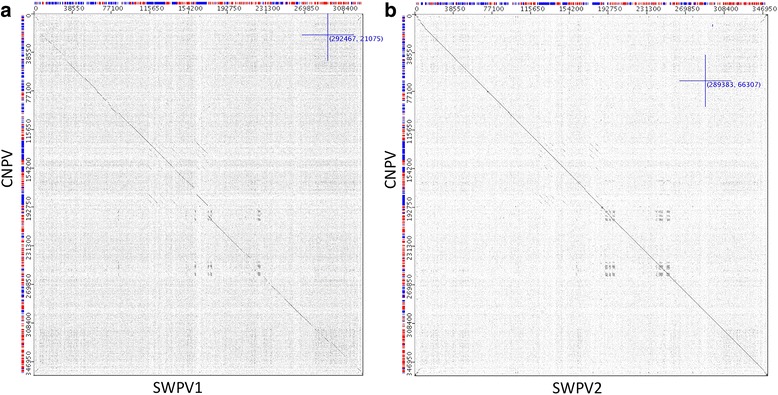
Dotplots of Shearwaterpox viruses (SWPV-1 and 2) vs CNPV genomes. Horizontal sequence: SWPV-1 (**a**) and SWPV-2 (**b**), vertical sequence CNPV. *Red* and *blue boxes* represent genes transcribed to the *right* and *left* of the genome, respectively

**Fig. 3:**
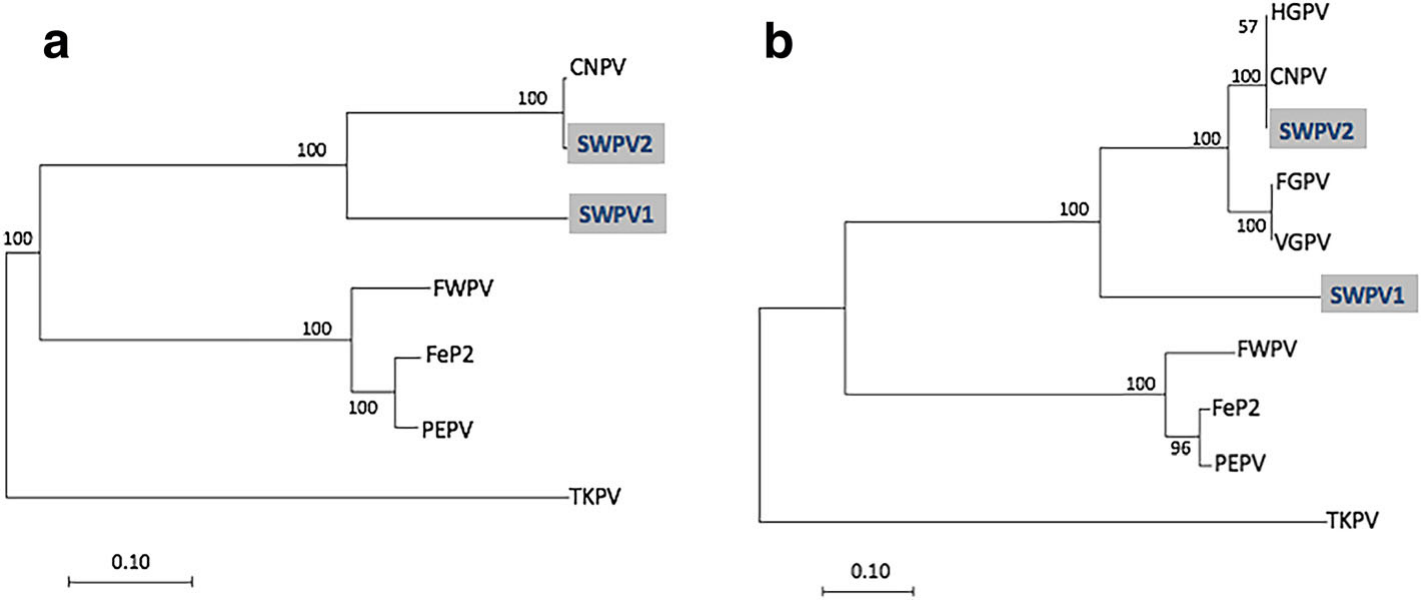
Phylogenetic relationship between Shearwaterpox viruses (SWPV-1 and 2) and other avipoxviruses. **a** Phylogenetic tree of 173 kbp core region (large gaps removed) from available complete avipoxvirus genomes. **b** Phylogenetic tree highlighting viruses closely related to CNPV. The sequences were aligned with ClustalO and MEGA7 was used to create a maximum likelihood tree based on the Tamura-Nei method and tested by bootstrapping with 1000 replicates. The abbreviations and GenBank accession details for poxviruses strains were used: *Canarypox virus* (CNPV; AY318871), *Pigeonpox virus* (FeP2; KJ801920), *Penguinpox virus* (PEPV; KJ859677) *Fowlpox virus* (FWPV; AF198100), *Shearwaterpox virus* 1 (SWPV-1; KX857216), *Shearwaterpox virus* 2 (SWPV-2; KX857215), *Turkeypox virus* (TKPV; NC_028238), *Vultur Gryphus poxvirus* (VGPV; AY246559), *Flamingopox virus* (FGPV; HQ875129 and KM974726), *Hawaiian goose poxvirus* (HGPV; AY255628)

**Fig. 4:**
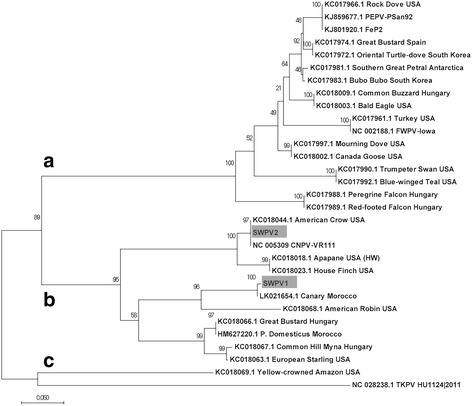
Maximum likelihood phylogenetic tree from partial DNA sequences of p4b gene of avipoxviruses. Novel Shearwaterpox viruses (SWPV-1 and SWPV-2) are highlighted by gray background

**Fig. 5:**
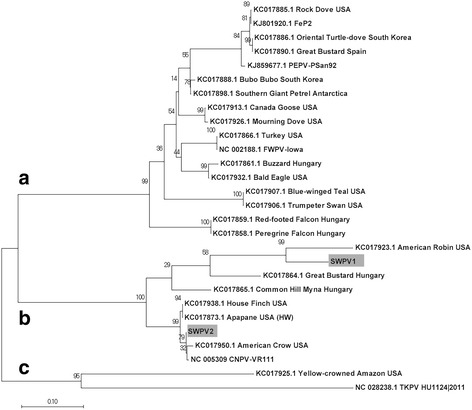
Maximum likelihood phylogenetic tree from partial DNA sequences of DNA polymerase gene of avipoxviruses. Novel Shearwaterpox viruses (SWPV-1 and SWPV-2) are highlighted by gray background

This difference in similarity between the new viruses and CNPV is easily visualized in complete genome dotplots (Fig. 2a and b). Significantly more indels are present in the SWPV-1 vs CNPV dotplot (Fig. 2a). However, when the phylogenetic relationships of these viruses were examined together with the other available complete genomes, SWPV-1 was still part of the CNPV clade (Fig. 3a). From this alignment, CNPV is 99.2%, 78.7%, 69.4%, 69.5%, 68.8% and 66.5% identical (nt) to SWPV-2, SWPV-1, FeP2, PEPV, FWPV and TKPV, respectively. A greater selection of viruses was included in the phylogenetic tree by using other fragments of incompletely sequenced avipoxvirus genomes. For example, *Vultur gryphus poxvirus* (VGPV), *Flamingopox virus* (FGPV) and *Hawaiian goose poxvirus* (HGPV) are all more similar to SWPV-2 and CNPV than SWPV-1 (Fig. 3b), this confirms that other poxviruses are as closely related to CNPV as SWPV-2. By also building phylogenetic trees with partial nucleotide sequences from the p4b gene (Fig. 4) and DNA polymerase gene (Fig. 5), we discovered that several other viruses are within the SWPV-1, SWPV-2 and CNPV clade. This includes a poxvirus isolated from Houbara Bustards (*Chlamydotis undulata*) in captive-breeding programs in Morocco [23], but named CNPV-morocco, and avipoxviruses isolated from American crow (*Corvus brachyrhynchos*) and American robin (*Turdus migratorius*) [24], which is almost identical to CPNV-1 within this relatively small fragment of the genome.

### Features of SWPV-2

As noted above, and displayed in the Dotplot (Fig. 2b), SWPV-2 is very similar to CNPV with almost 98% nt identity. However, a 1% difference still gives approximately 10 mutations in an average sized gene any of which could have drastic effects if an early STOP codon is introduced to the gene sequence. Similarly, small changes to promoter regions can significantly alter gene expressions that are impossible to predict in these viruses. With this annotation strategy, 18 CNPV genes were deemed to be missing from the SWPV-2 complete genome and a further 15 genes significantly fragmented as to probably cause them to be non-functional (Table 1). No novel genes were predicted in SWPV-2, and no rearrangement of genes compared to CNPV was observed.

### Features of SWPV-1

As expected from the much lower percent nt identity, SWPV-1 was found to be considerably more different to CNPV than SWPV-2 when compared at the level of genes present or absent. (Table 1). 43 CNPV genes are absent from SWPV-1 and a further 6 are significantly fragmented. There are 4 predicted genes in SWPV-1 that are not present in any other poxvirus, nor do they match any sequences in the NR protein database using BLASTP. However, they are all relatively short ORFs and it is possible that they are not functional genes. Additionally, SWPV-1 encodes nine polypeptides that do not match CNPV proteins, but do match proteins from other avipoxviruses (penguinpox, turkeypox, pigeonpox and fowlpox). This could be due to recombination among ancestral viruses, but could also result from the loss of the corresponding ortholog in CNPV leaving another virus to provide the “best match”.

As might be expected given the greater distance between SWPV-1 and CPNV than between SWPV-2 and CNPV, there are more instances of minor rearrangements that created a loss of synteny (Table 1). However, since most of these involve the families of repeated genes, it is also possible that divergence of these sequences has led to the inability to distinguish between the orthologous and paralogous genes.

### Evidence of recombination among avipoxviruses

**Fig. 6:**
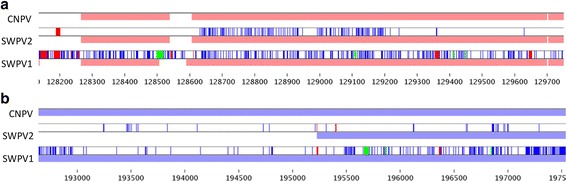
Region of recombination in Shearwaterpox viruses (SWPV) detected in *A. carneipes* and *A. pacificus*. Nucleotide differences to CNPV are shown in *blue* (SNPs), *green/red* (indels). Figure 6a. Region of recombination in SWPV-2. On the *middle track*, SWPV-2 has very few differences to CNPV except for highly divergent block in the middle of this region. Figure 6b. Region of recombination in SWPV-1. On the *bottom track*, SWPV-1 is very different to CNPV except for highly similar block between nt 193,000 and 195,500

When we reviewed a graph of nt identity between the 2 new complete genomes and CNPV using BBB (not shown), there were several relatively short syntenic regions where 1) SWPV-1 matched CNPV significantly better than the majority of the genome, and 2) SWPV-2 matched CNPV significantly worse than the majority of the genome. To examine these regions in more detail, the *Visual Summary* feature of BBB was used to display individual SNPs for these genome comparisons (Fig. 6a and b). This analysis revealed that SWPV-1 and SWPV-2 were unique in these regions and confirmed that the genome sequences of SWPV-1and SWPV-2 were not contaminated during their assembly. However, when these regions were used as query sequences in BLASTN searches of all poxvirus sequences the best match remained CNPV suggesting that these sequences originated from avipoxvirus genomes that are not represented in the public databases.

## Discussion

This paper describes the detection and characterization of two novel avipoxvirus complete genome sequences in a naturally occurring infections of avian pox in a naïve population of shearwaters. The DNA sequences of SWPV-1 and SWPV-2 are significantly different than each other but nevertheless had closest similarity with *Canarypox virus* (67% and 98%, respectively). Furthermore, the genetic distance and novel genome structure of SWPV-1 from *A. carneipes* considered to be missing 43 genes likened to CNPV and contained 4 predicted genes which are not found in any other poxvirus and is overall sufficiently genetically different to be considered a separate virus species. Whilst, the SWPV-2 complete genome was missing 18 genes compared to CNPV, with a further 15 genes significantly fragmented as to probably cause them to be non-functional. Furthermore, the phylogenetic distribution of SWPV-1 indicates that shearwaters and perhaps other long-lived, vagile marine birds could be important hosts for avipoxvirus dispersal around the globe. The natural hosts of these avipoxviruses maybe this population of shearwaters, other migratory birds that use Lord Howe Island for breeding or resident avian host reservoir species. Species such as the Lord Howe White-eye (*Zosterops tephropleura*) and Lord Howe Golden Whistler (*Pachcephala petoralis contempta*) are candidate passerine birds that might provide such function.

Examining the phylogenetic relationship between the *Shearwaterpox viruses* and other avipoxviruses, it is evident that the SWPV-2 is most closely related to *Canarypox virus*. The SWPV-1 and SWPV-2 complete genomes both contain several genes that are more closely related to CNPV throughout their entire genome. As shown in Fig. 3 it is reasonable to postulate that these viruses originated from a common ancestor that diverged from a CNPV-like progenitor related to f*owlpox*, *penguinpox* and *pigeonpox* viruses. Finer resolution of the phylogenetic relationship using partial nucleotide sequences of p4b and DNA polymerase genes of avipoxviruses revealed that SWPV isolated from seabirds also clustered in global clade B consisting of avipoxviruses originating from Canary Morocco, Canarypox and poxviruses from American crow and American robin. Given their genetic diversity, it is perhaps not surprising that Shearwater species can be exposed to multiple avipoxviral infections. Studies such as those by Barnett et al. [25] suggest that the species specificity of poxviruses is variable. Some genera, such as *Suipoxvirus* are highly restricted to individual vertebrate hosts, swinepox for instance, whereas others, such as avipoxviruses demonstrate some evidence of cross-species infection within a predator–prey system [24]. This suggests that the avipoxviruses can infect a diverse range of bird species if they are within a close enough proximity to each other [26]. Thus far, there were no clear patterns regarding species-specificity in the *Shearwaterpox viruses* described here.

While overt and systemic lesions and fatal disease can occur, avian pox tends to be a self-limiting localized infection of apterial skin with full recovery possible. Many bird species experience life-long immunity if the immune system is not weakened and or the birds are not infected by different strains [27, 28]. As shown in our example, secondary infections can occur and these may contribute to morbidity and mortality [29–31]. Similar to the example in shearwaters, Shivaprasad et al. [30] reported evidence of poxvirus infection and secondary fungal pathogens in canaries (*Serinus canaria*). Stressful conditions, poor nutrition, overt environmental contamination and other underlying causes of immunosuppression and ill health may contribute to the pathogenesis of such lesions. This was the primary reason we tested for avian circovirus and other potential pathogens.

Avian pox has not been previously reported in shearwaters (*Ardenna* spp.) from Lord Howe Island, nor has it been documented for any other bird species in this region. So it is difficult to attribute the causality of this unique event in these species. The value of complete genome characterization and analysis is highlighted since a phylogenetic relationship based on single gene studies such as the polymerase gene may have falsely implicated *Canarypox virus* as a potential exotic introduced emerging disease from domesticated birds. Although we cannot trace the actual source of infection in the shearwater chicks, it is more likely that the infection in the birds resulted from parental feeding or arthropod mediated transmission from other island bird species [32]. While, the reservoir host of these novel *Shearwaterpox viruses* is unknown, mosquitoes are suspected to play a part in transmission within the island. Avipoxvirus infection appears to be relatively rare in seabirds, but it has been reported in several species when they occur on human-inhabited islands that harbor mosquito vectors [33]. According to the Lord Howe Island Board, ship rats, mice, cats, humans and other invasive pest species such as owls are implicated in the extinction of at least five endemic birds, two reptiles, 49 flowering plants, 12 vegetation communities and numerous threatened invertebrates [34]. These rodents and invasive pests have also been highlighted for the potential reservoir of poxvirus infections [3, 35]. Transmission of avipoxvirus by prey–predator and other migratory seabirds likely plays a prominent role; however, the mode of avipoxvirus transmission on Lord Howe Island is not completely understood. Studies by Gyuranecz et al. [24], for example, postulated that raptors may acquire poxvirus infection from their avian prey. This suggests that the poxvirus in shearwaters is likely to be transmitted from other island species such as other migratory seabirds and/or prey–predator, although, it is difficult to be certain without further studies.

Interestingly, these new *shearwaterpox virus* complete genomes also provide evidence that supports the hypothesis that recombination may play an important role in the evolution of avipoxviruses. A number of genes in SWPV-1 appear to be rearranged compared to CNPV and blocks of unusual similarity scores were seen in both SWPVs. Software that is designed to look for gross recombination between two viruses, such as two strains of HIV, fails to detect this level of recombination and it is left to the investigator to observe such small events by eye after visualizing the distribution of SNPs between viruses. Such relatively small exchanges of DNA may still exert important influences on virus evolution, and has been predicted to have been a driver in the evolution of smallpox [36].

## Conclusions

These are the first avipoxvirus complete genome sequences that infect marine bird species. The novel complete genome sequences of SWPV-1 and −2 have greatly enhanced the genomic information for the *Avipoxvirus* genus, which will contribute to our understanding of the avipoxvirus more generally, and track the evolution of poxvirus infection in such a non-model avian species. Together with the sequence similarities observed between SWPV and other avipoxviruses, this study concluded that the SWPV complete genome from *A. carneipes* (SWPV-1) described here is not closely related to any other avipoxvirus complete genome isolated from avian or other natural host species, and that it likely should be considered a separate species. Further investigations of Shearwaterpox viruses genetic and pathogenesis will provide a unique approach to better assess the risk associated to poxvirus transmission within and between marine bird species.

## Methods

### Source of sampling

A total of six samples were collected from two different species of shearwater, five were from Flesh-footed Shearwater (ID: 15-1527-31), and other one was from Wedge-tailed Shearwater (ID: 15–1526). Of size birds, two were recoded to have evidence of gross well circumscribed lesions in the beak (Fig. 1a) and ankle, and others had feather defects (fault lines across the vanes of feathers). Samples were collected from fledglings (approximately 80–90 days of age) of both species on Lord Howe Island, New South Wales (32.53̊S, 159.08̊E) located approximately 500 km off the east coast of Australia during April-May 2015. Samples were collected with the permission of the Lord Howe Island Board (permit no. LHIB 02/14) under the approval of the University of Tasmania and Charles Sturt University Animal Ethics Committees (permit no. A0010874, A0011586, and 09/046). Samples from one individual of each shearwater species were collected including skin lesions, liver and skin biopsies, as well as blood for identifying the causative agents. Depending on the samples, either 25 mg of skin tissue were cut out and chopped into small pieces or 50–100 μL of blood were aseptically transferred into clean 1.5 mL microcentrifuge tube (Eppendorf), and genomic DNA was isolated using the Qiagen blood and tissue mini kit (Qiagen, Germany). The extracted DNA has been stored at −20 °C for further testing. Histopathological examination of the skin was performed.

### Archived viral and fungal pathogen testing

Initially, the extracted DNA was screened for detecting novel circoviruses [37, 38] and reticuloendotheliosis virus [39]. For poxvirus screening, the primers PoxP1 (5′-CAGCAGGTGCTAAACAACAA-3′) and PoxP2 (5′-CGGTAGCTTAACGCCGAATA-3′) were synthesized from published literature and used to amplify a segment of approximately 578 bp from the 4b core protein gene for all ChPV species [40]. Optimized PCR reactions mixture contained 3 μL of extracted genomic DNA, 25 pmol of each primer (GeneWorks, Australia), 1.5 mM MgCl_2,_ 1.25 mM of each dNTP, 1xGoTaq® Green Flexi Reaction Buffer, 1 U of Go Taq DNA polymerase (Promega Corporation, USA) and DEPC distilled H_2_O (Invitrogen, USA) was added to a final volume of 25 μL. The PCR amplification was carried out in an iCycler thermal cycler (Bio-Rad) under the following conditions: denaturation at 94 °C for 2 min followed by 35 cycles of 94 °C for 1 min, 60 °C for 1 min and 72 °C for 1 min, and a final extension step of 2 min at 72 °C.

The internal transcribed spacer (ITS) region was chosen for screening and identification of fungal pathogens [41]. A set of fungus-specific primers ITS1 (5′- TCCGTAGGTGAACCTGCGG -3′) and ITS4 (5′-TCCTCCGCTTATTGATATGC -3′) were designed and used to amplify a segment of approximately 550 bp from the fungal ITS gene [42]. The PCR was standardized to amplify ITS genes, and the 25-μL reaction mixture contained 3 μL of extracted genomic DNA, 25 pmol of each primer (GeneWorks, Australia), 1.5 mM MgCl_2,_ 1.25 mM of each dNTP, 1xGoTaq® Green Flexi Reaction Buffer, 1 U of Go Taq DNA polymerase (Promega Corporation, USA). The PCR reaction involved initial denaturation at 95 °C for 5 min, followed by 30 cycles of denaturation at 94 °C for 30 s, annealing at 58 °C for 30 s, and extension at 72 °C for 1 min, and with a final step of one cycle extension at 72 °C for 10 min.

Amplified PCR products, together with a standard molecular mass marker (Sigma), were separated by electrophoresis in 2.0% agarose gel and stained with GelRed (Biotium, CA). Selected bands were excised and purified using the Wizard® SV Gel and PCR Clean-Up System (Promega, USA) according to the manufacturer’s instructions. Purified amplicons were sequenced with PCR primers by the Australian Genome Research Facility Ltd (Sydney) using an AB 3730xl unit (Applied Biosystems). For each amplicon, sequences were obtained at least twice in each direction for each isolate. The sequences were trimmed for primers and aligned to construct contigs (minimum overlap of 35 bp, minimum match percentage of 95%) using Geneious Pro (version 10.0.2).

### High throughput sequencing

Next-generation sequencing (NGS) was used to sequence the poxvirus genomes. Virion enrichment was performed by centrifugation for 2 min at 800 × g to remove tissue debris, and the supernatants were subsequently filtered through 5 μm centrifuge filters (Millipore) [43]. The filtrates were nuclease treated to remove unprotected nucleic acids using 8 μL RNase Cocktail Enzyme Mix (Invitrogen). Viral nucleic acids were subsequently extracted using QIAamp DNA mini (Qiagen). The genomic libraries were prepared with an insert size of 150 paired-end. DNA sequencing (NGS) was performed on a HiSeq4000 sequencing platform (Illumina) by Novogene, China.

### Bioinformatics

Assembly of the viral genome was conducted according to the established pipeline [44] in CLC Genomics workbench 9.5.2 under La Trobe University Genomics Platform. Briefly, the preliminary quality evaluation for each raw read was generated using quality control (QC) report. The raw data were preprocessed to remove ambiguous base calls, and bases or entire reads of poor quality using default parameters. The datasets were trimmed to pass the quality control based on PHRED score or per base sequence quality score. Trimmed sequence reads were mapped against closely available host genome (Albatross) to remove possible remaining host DNA contamination, and post-filtered reads were mapped against reference *Canarypox virus* complete genome sequence. Consensus sequences were used to generate the complete poxvirus genome. Avipoxvirus complete genome sequences were aligned using MAFFT [45]. Then the poxvirus specific bioinformatics analyses were performed using the Viral Bioinformatics Resource Centre (virology.uvic.ca) [46], and the further analyses were conducted using the following tools: Viral Orthologous Clusters Database for sequence management (VOCs) [11]; Base-By-Base for genome/gene/protein alignments [47, 48]; Viral Genome Organizer for genome organization comparisons (VGO) [11], and Genome Annotation Transfer Utility for annotation (GATU) [49].

Open reading frames (ORFs) longer than 60 amino acids with minimal overlapping (overlaps cannot exceed 25% of one of the genes) to other ORFs were captured using the CLC Genomics Workbench (CLC) ORF analysis tool as well as GATU [49], and other protein coding sequence and annotation software described in Geneious (version 10.0.2, Biomatters, New Zealand). These ORFs were subsequently extracted into a FASTA file, and similarity searches including nucleotide (BLASTN) and protein (BLASTP) were performed on annotated ORFs as potential genes if they shared significant sequence similarity to known viral or cellular genes (BLAST E value ≤ e-5) or contained a putative conserved domain as predicted by BLASTp [50]. The final SWPV annotation was further examined with other poxvirus ortholog alignments to determine the correct methionine start site, correct stop codons, signs of truncation, and validity of overlaps.

### Phylogenetic analysis

Phylogenetic analyses were performed using full poxvirus genome sequences for Shearwater species determined in this study with related avipoxvirus genome sequences available in GenBank database. A selection of partial sequences from seven completely sequenced avipoxvirus genomes and fragments of incompletely sequenced avipoxvirus genomes from Vultur Gryphus poxvirus (VGPV), flamingopox virus (FGPV) and Hawaiian goose poxvirus (HGPV) were also used for phylogenetic analysis. To investigate closer evolutionary relationship among avipoxviruses, partial nucleotide sequences of p4b and DNA polymerase genes were selected. The avipoxvirus sequences were aligned using ClustalO, and then manually edited in Base-by-Base. MEGA7 was used to create a maximum likelihood tree based on the Tamura-Nei method and tested by bootstrapping with 1000 replicates. An additional analysis was performed using complete genome nucleotide sequences of *Canarypox virus* (CNPV; AY318871), *Pigeonpox virus* (FeP2; KJ801920), *Fowlpox virus* (FPV; AF198100), *Turkeypox virus* (TKPV; NC_028238), *Shearwaterpox virus* strain-1 (SWPV-1; KX857216), and *Shearwaterpox virus* strain-2 (SWPV-2; KX857215), which were aligned with MAFTT in Base-By-Base for genome/gene/protein alignments [48]. The program jModelTest 2.1.3 favoured a general-time-reversible model with gamma distribution rate variation and a proportion of invariable sites (GTR + I + G4) for the ML analysis [51]*.*

## Additional files

Additional file 1: Table S1. Summary of SWPV1 genome annotations (DOCX 52 kb)

Additional file 2: Table S2. Summary of SWPV2 genome annotations (DOCX 145 kb)

## Abbreviations

ChPV: Chordopoxvirinae
CNPV: *Canarypox virus*
dsDNA: double-stranded
FGPV: *Flamingopox virus*
FP9: European strain of *Fowlpox virus*
FPVUS: South African strain *of Fowlpox virus*
GATU: Genome Annotation Transfer Utility
HGPV: *Hawaiian goose poxvirus*
ITS: internal transcribed spacer
ML: Maximum likelihood
NGS: Next-generation sequencing
ORF: open reading frame
PCR: polymerase chain reaction
PEPV: *Penguinpox virus*
QC: Quality control
SWPV-1: Shearwaterpox virus 1
SWPV-2: Shearwaterpox virus 2
TKPV: *Turkeypox virus*
