# Supplementary material for: Genomic characterization of two novel pathogenic avipoxviruses isolated from pacific shearwaters (Ardenna spp.)
Source: BMC Genomics. 2017 Apr 13;18:298. doi: 10.1186/s12864-017-3680-z (PMC5390406; doi:10.1186/s12864-017-3680-z)
Supplement: Supplementary file 1 — Summary of SWPV1 genome annotations (DOCX 52 kb) [file 12864_2017_3680_MOESM1_ESM.docx]

**Table S1** Summary of Shearwaterpox virus-1 (SWPV1) genome annotations

| **SWPV1 Annotation Table using CNPV as reference** | | | | |
| --- | --- | --- | --- | --- |
| **Name of SWPV gene** | **ORF position** | **#AA** | **Counterpart** | **Annotation Special Notes** |
| SWPV1-001 | 638-1183 | 181 | CNPV003 C-type lectin-like protein |  |
| SWPV1-002 | 1695-3101 | 468 | CNPV004 ankyrin repeat protein |  |
| SWPV1-003 | 3227-3889 | 220 | CNPV005 conserved hypothetical protein |  |
| SWPV1-004 | 4406-4930 | 174 | CNPV008 C-type lectin-like protein |  |
| SWPV1-005 | 5107-5523 | 138 | CNPV035 C-type lectin-like protein |  |
| SWPV1-006 | 6103-7566 | 487 | CNPV318 ankyrin repeat protein |  |
| SWPV1-007 | 7663-8016 | 117 | CNPV016 C-type lectin-like protein |  |
| SWPV1-008 | 8267-9544 | 425 | CNPV017 ankyrin repeat protein |  |
| SWPV1-009 | 9739-10572 | 277 | CNPV295 ankyrin repeat protein |  |
| SWPV1-010 | 12030-13268 | 412 | CNPV020 ankyrin repeat protein |  |
| SWPV1-011 | 13265-14716 | 483 | CNPV320 Ig-like domain protein |  |
| SWPV1-012 | 14716-16302 | 528 | CNPV021 ankyrin repeat protein |  |
| SWPV1-013 | 16330-17400 | 356 | CNPV022 putative serpin |  |
| SWPV1-014 | 17486-18059 | 190 | CNPV309 ankyrin repeat protein |  |
| SWPV1-015 | 18161-19753 | 530 | CNPV310 ankyrin repeat protein |  |
| SWPV1-016 | 19829-20743 | 304 | CNPV025 alpha-SNAP-like protein |  |
| SWPV1-017 | 20825-22018 | 397 | CNPV026 ankyrin repeat protein |  |
| SWPV1-018 | 22070-24010 | 646 | CNPV027 ankyrin repeat protein |  |
| SWPV1-019 | 24098-25324 | 408 | CNPV028 ankyrin repeat protein |  |
| SWPV1-020 | 25443-25871 | 142 | CNPV029 C-type lectin-like protein |  |
| SWPV1-021 | 25907-26944 | 345 | CNPV030 ankyrin repeat protein |  |
| SWPV1-022 | 27145-27507 | 120 | CNPV031 hypothetical protein |  |
| SWPV1-023 | 27706-28212 | 168 | CNPV013 conserved hypothetical protein |  |
| SWPV1-024 | 28290-29021 | 243 | CNPV032 Ig-like domain putative IFN-gamma binding protein |  |
| SWPV1-025 | 29147-29863 | 238 | CNPV033 Ig-like domain protein |  |
| SWPV1-026 | 30119-30385 | 88 | CNPV036 conserved hypothetical protein |  |
| SWPV1-027 | 30446-30982 | 178 | CNPV037 conserved hypothetical protein |  |
| SWPV1-028 | 30997-32232 | 411 | CNPV038 vaccinia C4L/C10L-like protein |  |
| SWPV1-029 | 32339-33310 | 323 | CNPV039 G protein-coupled receptor-like protein |  |
| SWPV1-030 | 33324-35093 | 589 | CNPV040 ankyrin repeat protein |  |
| SWPV1-031 | 35149-36447 | 432 | CNPV041 ankyrin repeat protein |  |
| SWPV1-032 | 36492-38318 | 608 | CNPV042 ankyrin repeat protein |  |
| SWPV1-033 | 38384-38992 | 202 | CNPV043 conserved hypothetical protein |  |
| SWPV1-034 | 39033-40499 | 488 | CNPV044 ankyrin repeat protein |  |
| SWPV1-035 | 40682-41677 | 331 | CNPV045 G protein-coupled receptor-like protein |  |
| SWPV1-036 | 41702-43060 | 452 | CNPV046 ankyrin repeat protein |  |
| SWPV1-037 | 43123-43500 | 125 | CNPV047 conserved hypothetical protein |  |
| SWPV1-038 | 43698-46112 | 804 | CNPV048 alkaline phosphodiesterase-like protein |  |
| SWPV1-039 | 46196-46642 | 148 | CNPV049 hypothetical protein |  |
| SWPV1-040 | 46678-47736 | 352 | CNPV050 ankyrin repeat protein |  |
| SWPV1-041 | 47782-48978 | 398 | CNPV051 DNase II-like protein |  |
| SWPV1-042 | 49035-49583 | 182 | CNPV052 C-type lectin-like protein |  |
| SWPV1-043 | 49643-50632 | 329 | CNPV017 ankyrin repeat protein |  |
| SWPV1-044 | 50756-51163 | 135 | CNPV053 conserved hypothetical protein |  |
| SWPV1-045 | 51234-51659 | 141 | CNPV054 conserved hypothetical protein |  |
| SWPV1-046 | 51709-52197 | 162 | CNPV055 conserved hypothetical protein |  |
| SWPV1-047 | 52194-52661 | 155 | CNPV056 dUTPase |  |
| SWPV1-048 | 52699-53604 | 301 | CNPV057 putative serpin |  |
| SWPV1-049 | 53643-54167 | 174 | CNPV058 bcl-2 like protein |  |
| SWPV1-050 | 54232-55248 | 338 | CNPV059 putative serpin |  |
| SWPV1-051 | 55311-56021 | 236 | CNPV060 conserved hypothetical protein |  |
| SWPV1-052 | 56116-57819 | 567 | CNPV061 DNA ligase |  |
| SWPV1-053 | 57853-58902 | 349 | CNPV062 putative serpin |  |
| SWPV1-054 | 58970-60049 | 359 | CNPV063 hydroxysteroid dehydrogenase-like protein |  |
| SWPV1-055 | 60103-60921 | 272 | CNPV064 TGF-beta-like protein |  |
| SWPV1-056 | 61048-62769 | 573 | CNPV065 semaphorin-like protein |  |
| SWPV1-057 | 62870-63289 | 139 | CNPV066 hypothetical protein |  |
| SWPV1-058 | 63372-63542 | 56 | No Ortholog |  |
| SWPV1-059 | 63676-64449 | 257 | CNPV068 GNS1/SUR4-like protein |  |
| SWPV1-060 | 64533-64997 | 154 | CNPV069 late transcription factor VLTF-2 |  |
| SWPV1-061 | 65014-66675 | 553 | CNPV070 putative rifampicin resistance protein, IMV assembly |  |
| SWPV1-062 | 66701-67570 | 289 | CNPV071 mRNA capping enzyme small subunit |  |
| SWPV1-063 | 67772-68104 | 110 | CNPV073 hypothetical protein |  |
| SWPV1-064 | 68219-70126 | 635 | CNPV074 NPH-I, transcription termination factor |  |
| SWPV1-065 | 70123-70803 | 226 | CNPV075 mutT motif putative gene expression regulator |  |
| SWPV1-066 | 70787-71488 | 233 | CNPV076 mutT motif |  |
| SWPV1-067 | 71633-72940 | 435 | CNPV011 ankyrin repeat protein |  |
| SWPV1-068 | 73923-74408 | 161 | CNPV078 RNA polymerase subunit RPO18 |  |
| SWPV1-069 | 74395-76296 | 633 | CNPV080 early transcription factor small subunit VETFS |  |
| SWPV1-070 | 76277-78649 | 790 | CNPV082 NTPase, DNA replication |  |
| SWPV1-071 | 78809-79480 | 223 | CNPV083 CC chemokine-like protein |  |
| SWPV1-072 | 79648-80235 | 195 | CNPV215 CC chemokine-like protein |  |
| SWPV1-073 | 80260-80922 | 220 | CNPV084 uracil DNA glycosylase |  |
| SWPV1-074 | 80922-81659 | 245 | CNPV085 putative RNA phosphatase |  |
| SWPV1-075 | 81697-82893 | 398 | CNPV216 conserved hypothetical protein |  |
| SWPV1-076 | 83007-83363 | 118 | CNPV086 TNFR-like protein |  |
| SWPV1-077 | 83507-84277 | 256 | CNPV227 N1R/p28-like protein |  |
| SWPV1-078 | 85362-85676 | 104 | CNPV088 conserved hypothetical protein |  |
| SWPV1-079 | 85651-86145 | 164 | CNPV089 conserved hypothetical protein |  |
| SWPV1-080 | 86132-86506 | 124 | CNPV090 conserved hypothetical protein |  |
| SWPV1-081 | 86574-86807 | 77 | CNPV091 HT motif protein |  |
| SWPV1-082 | 87004-87426 | 140 | CNPV092 conserved hypothetical protein |  |
| SWPV1-083 | 87513-88325 | 270 | CNPV093 virion protein |  |
| SWPV1-084 | 88384-89232 | 282 | CNPV094 T10-like protein |  |
| SWPV1-085 | 89218-89361 | 47 | CNPV095 conserved hypothetical protein |  |
| SWPV1-086 | 89373-89606 | 77 | CNPV096 ubiquitin |  |
| SWPV1-087 | 89724-90620 | 298 | CNPV097 conserved hypothetical protein |  |
| SWPV1-088 | 90627-90812 | 61 | CNPV098 hypothetical protein |  |
| SWPV1-089 | 90823-91383 | 186 | CNPV099 beta-NGF-like protein |  |
| SWPV1-090 | 91401-92036 | 211 | CNPV100 putative interleukin binding protein |  |
| SWPV1-091 | 92171-92479 | 102 | CNPV102 conserved hypothetical protein |  |
| SWPV1-092 | 92498-93064 | 188 | CNPV103 N1R/p28-like protein |  |
| SWPV1-093 | 93254-93631 | 125 | CNPV104 putative glutaredoxin 2, virion morphogenesis |  |
| SWPV1-094 | 93574-94278 | 234 | CNPV106 putative elongation factor |  |
| SWPV1-095 | 94272-94583 | 103 | CNPV105 conserved hypothetical protein |  |
| SWPV1-096 | 94612-95946 | 444 | CNPV162 TGF-beta-like protein |  |
| SWPV1-097 | 95995-97896 | 633 | CNPV108 putative metalloprotease, virion morphogenesis |  |
| SWPV1-098 | 97880-99925 | 681 | CNPV109 NPH-II, RNA helicase |  |
| SWPV1-099 | 99960-101225 | 421 | CNPV110 virion core proteinase |  |
| SWPV1-100 | 101230-102405 | 391 | CNPV111 DNA-binding protein |  |
| SWPV1-101 | 102406-102651 | 81 | CNPV112 putative IMV membrane protein |  |
| SWPV1-102 | 102675-103220 | 181 | CNPV113 thymidine kinase |  |
| SWPV1-103 | 103303-103542 | 79 | CNPV114 HT motif protein |  |
| SWPV1-104 | 103622-104503 | 293 | CNPV115 DNA-binding phosphoprotein |  |
| SWPV1-105 | 104504-104704 | 66 | CNPV116 unnamed protein product |  |
| SWPV1-106 | 104711-105655 | 314 | CNPV117 DNA-binding virion protein |  |
| SWPV1-107 | 105845-107815 | 656 | CNPV118 conserved hypothetical protein |  |
| SWPV1-108 | 107745-108140 | 131 | CNPV119 virion core protein |  |
| SWPV1-109 | 108137-108421 | 94 | CNPV120 putative IMV redox protein, virus assembly |  |
| SWPV1-110 | 108448-111414 | 988 | CNPV121 DNA polymerase |  |
| SWPV1-111 | 111406-112227 | 273 | CNPV122 putative membrane protein |  |
| SWPV1-112 | 112229-113944 | 571 | CNPV123 conserved hypothetical protein |  |
| SWPV1-113 | 113978-119698 | 1906 | CNPV124 variola B22R-like protein |  |
| SWPV1-114 | 119784-125012 | 1742 | CNPV125 variola B22R-like protein |  |
| SWPV1-115 | 125198-130906 | 1902 | CNPV126 variola B22R-like protein |  |
| SWPV1-116 | 131002-131550 | 182 | CNPV127 RNA polymerase subunit RPO30 |  |
| SWPV1-117 | 131592-133820 | 742 | CNPV128 conserved hypothetical protein |  |
| SWPV1-118 | 133756-135174 | 472 | CNPV129 poly(A) polymerase large subunit PAPL |  |
| SWPV1-119 | 135168-135512 | 114 | CNPV130 DNA-binding virion core protein |  |
| SWPV1-120 | 135588-136226 | 212 | CNPV131 conserved hypothetical protein |  |
| SWPV1-121 | 136324-136779 | 151 | CNPV132 conserved hypothetical protein |  |
| SWPV1-122 | 136950-137222 | 90 | CNPV133 conserved hypothetical protein |  |
| SWPV1-123 | 137289-142700 | 1803 | CNPV134 variola B22R-like protein |  |
| SWPV1-124 | 142768-143904 | 378 | CNPV135 putative palmitylated EEV envelope lipase |  |
| SWPV1-125 | 143943-145811 | 622 | CNPV136 putative EEV maturation protein |  |
| SWPV1-126 | 145838-147241 | 467 | CNPV137 conserved hypothetical protein |  |
| SWPV1-127 | 147350-148687 | 445 | CNPV138 putative serine/threonine protein kinase, virus assembly |  |
| SWPV1-128 | 148662-149303 | 213 | CNPV139 conserved hypothetical protein |  |
| SWPV1-129 | 149382-149579 | 65 | CNPV140 conserved hypothetical protein |  |
| SWPV1-130 | 149878-150426 | 182 | CNPV141 HAL3-like domain protein |  |
| SWPV1-131 | 150564-150869 | 101 | No Ortholog |  |
| SWPV1-132 | 151153-152097 | 314 | CNPV142 N1R/p28-like protein |  |
| SWPV1-133 | 152225-154129 | 634 | CNPV143 ankyrin repeat protein |  |
| SWPV1-134 | 154161-155849 | 562 | CNPV144 ankyrin repeat protein |  |
| SWPV1-135 | 156527-157846 | 439 | CNPV145 conserved hypothetical protein |  |
| SWPV1-136 | 157856-158056 | 66 | CNPV146 RNA polymerase subunit RPO7 |  |
| SWPV1-137 | 158056-158622 | 188 | CNPV147 conserved hypothetical protein |  |
| SWPV1-138 | 158587-159630 | 347 | CNPV148 virion core protein |  |
| SWPV1-139 | 160086-161114 | 342 | CNPV159 N1R/p28-like protein |  |
| SWPV1-140 | 161259-161540 | 93 | CNPV232 CC chemokine-like protein |  |
| SWPV1-141 | 161685-167504 | 1939 | CNPV154 variola B22R-like protein | Low SNP Density |
| SWPV1-142 | 167553-172985 | 1810 | CNPV155 variola B22R-like protein |  |
| SWPV1-143 | 173999-175036 | 345 | CNPV166 Ig-like domain protein | Low SNP Density |
| SWPV1-144 | 175311-175829 | 172 | CNPV167 Ig-like domain protein | Low SNP Density |
| SWPV1-145 | 175922-176935 | 337 | CNPV169 N1R/p28-like protein | CNPV-168/169 Fusion |
| SWPV1-146 | 177008-177352 | 114 | CNPV170 thymidylate kinase | Fragment |
| SWPV1-147 | 177682-178464 | 260 | CNPV171 late transcription factor VLTF-1 |  |
| SWPV1-148 | 178478-179488 | 336 | CNPV172 putative myristylated protein |  |
| SWPV1-149 | 179489-180220 | 243 | CNPV173 putative myristylated IMV envelope protein |  |
| SWPV1-150 | 180262-180552 | 96 | CNPV174 conserved hypothetical protein |  |
| SWPV1-151 | 180542-181453 | 303 | CNPV175 conserved hypothetical protein |  |
| SWPV1-152 | 181479-182240 | 253 | CNPV176 DNA-binding virion core protein |  |
| SWPV1-153 | 182241-182636 | 131 | CNPV177 conserved hypothetical protein |  |
| SWPV1-154 | 182590-183036 | 148 | CNPV178 putative IMV membrane protein |  |
| SWPV1-155 | 183069-183971 | 300 | CNPV179 poly(A) polymerase small subunit PAPS |  |
| SWPV1-156 | 183968-184528 | 186 | CNPV180 RNA polymerase subunit RPO22 |  |
| SWPV1-157 | 184521-184931 | 136 | CNPV181 conserved hypothetical protein |  |
| SWPV1-158 | 184975-188841 | 1288 | CNPV182 RNA polymerase subunit RPO147 |  |
| SWPV1-159 | 188850-189350 | 166 | CNPV183 putative protein-tyrosine phosphatase, virus assembly |  |
| SWPV1-160 | 189366-189938 | 190 | CNPV184 conserved hypothetical protein |  |
| SWPV1-161 | 189927-190940 | 337 | CNPV185 ankyrin repeat protein |  |
| SWPV1-162 | 190972-191961 | 329 | CNPV186 IMV envelope protein |  |
| SWPV1-163 | 192034-194433 | 799 | CNPV187 RNA polymerase associated protein RAP94 |  |
| SWPV1-164 | 194582-195094 | 170 | CNPV188 late transcription factor VLTF-4 |  |
| SWPV1-165 | 195095-196045 | 316 | CNPV189 DNA topoisomerase |  |
| SWPV1-166 | 196050-196511 | 153 | CNPV190 conserved hypothetical protein |  |
| SWPV1-167 | 196474-196785 | 103 | CNPV191 conserved hypothetical protein |  |
| SWPV1-168 | 196793-199339 | 848 | CNPV192 mRNA capping enzyme large subunit |  |
| SWPV1-169 | 199387-199728 | 113 | CNPV193 HT motif protein |  |
| SWPV1-170 | 199725-200144 | 139 | CNPV194 virion protein |  |
| SWPV1-171 | 200220-200639 | 139 | CNPV195 hypothetical protein |  |
| SWPV1-172 | 200708-201277 | 189 | CNPV196 conserved hypothetical protein |  |
| SWPV1-173 | 201357-202196 | 279 | CNPV197 N1R/p28-like protein |  |
| SWPV1-174 | 202267-202746 | 159 | CNPV198 C-type lectin-like protein |  |
| SWPV1-175 | 202995-203663 | 222 | CNPV199 deoxycytidine kinase-like protein |  |
| SWPV1-176 | 203685-204143 | 152 | CNPV200 Rep-like protein |  |
| SWPV1-177 | 204246-204839 | 197 | CNPV201 conserved hypothetical protein |  |
| SWPV1-178 | 204892-205719 | 275 | CNPV202 N1R/p28-like protein |  |
| SWPV1-179 | 205772-206914 | 380 | CNPV203 N1R/p28-like protein |  |
| SWPV1-180 | 206976-207137 | 53 | CNPV204 conserved hypothetical protein |  |
| SWPV1-181 | 207309-208262 | 317 | CNPV205 N1R/p28-like protein |  |
| SWPV1-182 | 208322-209716 | 464 | CNPV206 putative photolyase |  |
| SWPV1-183 | 210050-211048 | 332 | CNPV081 Ig-like domain protein |  |
| SWPV1-184 | 211191-211772 | 193 | CNPV207 N1R/p28-like protein |  |
| SWPV1-185 | 211810-212328 | 172 | CNPV208 conserved hypothetical protein |  |
| SWPV1-186 | 212368-213303 | 311 | CNPV209 N1R/p28-like protein |  |
| SWPV1-187 | 213353-213745 | 130 | CNPV210 N1R/p28-like protein |  |
| SWPV1-188 | 213804-213968 | 54 | CNPV211 conserved hypothetical protein |  |
| SWPV1-189 | 214019-214552 | 177 | CNPV212 N1R/p28-like protein |  |
| SWPV1-190 | 214549-214761 | 70 | No Ortholog |  |
| SWPV1-191 | 214784-215434 | 216 | CNPV213 deoxycytidine kinase-like protein |  |
| SWPV1-192 | 215533-216030 | 165 | CNPV012 conserved hypothetical protein |  |
| SWPV1-193 | 216111-218135 | 674 | CNPV223 ankyrin repeat protein |  |
| SWPV1-194 | 218281-218889 | 202 | CNPV215 CC chemokine-like protein |  |
| SWPV1-195 | 220279-221400 | 373 | CNPV223 ankyrin repeat protein | Fragment |
| SWPV1-196 | 221557-222513 | 318 | CNPV218 N1R/p28-like protein |  |
| SWPV1-197 | 222678-223163 | 161 | CNPV160 N1R/p28-like protein | Fragment |
| SWPV1-198 | 223224-224327 | 367 | CNPV160 N1R/p28-like protein | Fragment/CNPV-220/221 Fusion |
| SWPV1-199 | 224375-225457 | 360 | CNPV160 N1R/p28-like protein |  |
| SWPV1-200 | 225663-226433 | 256 | CNPV161 TGF-beta-like protein |  |
| SWPV1-201 | 226494-226919 | 141 | CNPV162 TGF-beta-like protein |  |
| SWPV1-202 | 226954-227250 | 98 | No Ortholog |  |
| SWPV1-203 | 227747-228127 | 126 | CNPV224 hypothetical protein |  |
| SWPV1-204 | 228143-229414 | 423 | CNPV229 ankyrin repeat protein |  |
| SWPV1-205 | 229553-229855 | 100 | CNPV231 MyD116-like domain protein | Fragment |
| SWPV1-206 | 229924-230541 | 205 | CNPV232 CC chemokine-like protein |  |
| SWPV1-207 | 230618-232048 | 476 | CNPV233 ankyrin repeat protein |  |
| SWPV1-208 | 232534-233796 | 420 | CNPV214 vaccinia C4L/C10L-like protein |  |
| SWPV1-209 | 233939-234913 | 324 | CNPV236 ribonucleotide reductase small subunit |  |
| SWPV1-210 | 234967-236646 | 559 | CNPV234 ankyrin repeat protein |  |
| SWPV1-211 | 236665-237342 | 225 | CNPV238 late transcription factor VLTF-3 |  |
| SWPV1-212 | 237339-237557 | 72 | CNPV239 virion redox protein |  |
| SWPV1-213 | 237571-239553 | 660 | CNPV240 virion core protein P4b |  |
| SWPV1-214 | 239637-240365 | 242 | CNPV241 immunodominant virion protein |  |
| SWPV1-215 | 240404-240913 | 169 | CNPV242 RNA polymerase subunit RPO19 |  |
| SWPV1-216 | 240908-242029 | 373 | CNPV243 conserved hypothetical protein |  |
| SWPV1-217 | 242036-244165 | 709 | CNPV244 early transcription factor large subunit VETFL |  |
| SWPV1-218 | 244230-245132 | 300 | CNPV245 intermediate transcription factor VITF-3 |  |
| SWPV1-219 | 245094-245324 | 76 | CNPV246 putative IMV membrane protein |  |
| SWPV1-220 | 245325-248018 | 897 | CNPV247 virion core protein P4a |  |
| SWPV1-221 | 248035-248880 | 281 | CNPV248 conserved hypothetical protein |  |
| SWPV1-222 | 248877-249380 | 167 | CNPV249 virion protein |  |
| SWPV1-223 | 249395-249616 | 73 | CNPV250 conserved hypothetical protein |  |
| SWPV1-224 | 249602-249811 | 69 | CNPV251 putative IMV membrane protein |  |
| SWPV1-225 | 249858-250136 | 92 | CNPV252 putative IMV membrane protein |  |
| SWPV1-226 | 250153-250314 | 53 | CNPV253 putative IMV membrane virulence factor |  |
| SWPV1-227 | 250330-250620 | 96 | CNPV254 conserved hypothetical protein |  |
| SWPV1-228 | 250604-251710 | 368 | CNPV255 predicted myristylated protein |  |
| SWPV1-229 | 251726-252292 | 188 | CNPV256 putative phosphorylated IMV membrane protein |  |
| SWPV1-230 | 252310-253698 | 462 | CNPV257 DNA helicase, transcriptional elongation |  |
| SWPV1-231 | 253666-253926 | 86 | CNPV258 conserved hypothetical protein |  |
| SWPV1-232 | 253934-254272 | 112 | CNPV259 DNA polymerase processivity factor |  |
| SWPV1-233 | 254271-255569 | 432 | CNPV260 conserved hypothetical protein |  |
| SWPV1-234 | 255566-256021 | 151 | CNPV261 Holliday junction resolvase protein |  |
| SWPV1-235 | 256047-257198 | 383 | CNPV262 intermediate transcription factor VITF-3 |  |
| SWPV1-236 | 257264-260740 | 1158 | CNPV263 RNA polymerase subunit RPO132 |  |
| SWPV1-237 | 260729-262537 | 602 | CNPV264 A type inclusion-like protein |  |
| SWPV1-238 | 262572-263987 | 471 | CNPV265 A type inclusion-like/fusion protein |  |
| SWPV1-239 | 263988-264410 | 140 | CNPV266 conserved hypothetical protein |  |
| SWPV1-240 | 264429-265340 | 303 | CNPV267 RNA polymerase subunit RPO35 |  |
| SWPV1-241 | 265315-265533 | 72 | CNPV268 conserved hypothetical protein |  |
| SWPV1-242 | 265687-266028 | 113 | CNPV269 conserved hypothetical protein |  |
| SWPV1-243 | 266036-266395 | 119 | CNPV270 conserved hypothetical protein |  |
| SWPV1-244 | 266392-267210 | 272 | CNPV271 DNA packaging protein |  |
| SWPV1-245 | 267315-267863 | 182 | CNPV272 C-type lectin-like EEV protein |  |
| SWPV1-246 | 267895-268413 | 172 | CNPV012 conserved hypothetical protein |  |
| SWPV1-247 | 268462-269292 | 276 | CNPV273 conserved hypothetical protein |  |
| SWPV1-248 | 269337-270197 | 286 | CNPV274 putative tyrosine protein kinase |  |
| SWPV1-249 | 270204-271226 | 340 | CNPV275 putative serpin |  |
| SWPV1-250 | 271220-271903 | 227 | CNPV276 conserved hypothetical protein |  |
| SWPV1-251 | 272011-272943 | 310 | CNPV277 G protein-coupled receptor-like protein |  |
| SWPV1-252 | 272954-273247 | 97 | CNPV278 conserved hypothetical protein |  |
| SWPV1-253 | 273306-273809 | 167 | CNPV279 beta-NGF-like protein |  |
| SWPV1-254 | 273812-274216 | 134 | CNPV280 HT motif protein |  |
| SWPV1-255 | 274317-274895 | 192 | CNPV281 conserved hypothetical protein |  |
| SWPV1-256 | 274916-275272 | 118 | CNPV282 HT motif protein |  |
| SWPV1-257 | 275360-275692 | 110 | CNPV283 CC chemokine-like protein |  |
| SWPV1-258 | 275801-276379 | 192 | CNPV284 putative interleukin binding protein |  |
| SWPV1-259 | 276424-276795 | 123 | CNPV285 EGF-like protein |  |
| SWPV1-260 | 276798-277709 | 303 | CNPV286 putative serine/threonine protein kinase |  |
| SWPV1-261 | 277752-278249 | 165 | CNPV287 conserved hypothetical protein |  |
| SWPV1-262 | 278294-278785 | 163 | CNPV288 C-type lectin-like protein |  |
| SWPV1-263 | 278814-279212 | 132 | CNPV289 putative interleukin binding protein |  |
| SWPV1-264 | 279278-279505 | 75 | CNPV290 conserved hypothetical protein |  |
| SWPV1-265 | 279570-281435 | 621 | CNPV291 ankyrin repeat protein |  |
| SWPV1-266 | 281446-281751 | 101 | CNPV292 hypothetical protein |  |
| SWPV1-267 | 281726-282643 | 305 | CNPV293 ankyrin repeat protein |  |
| SWPV1-268 | 282694-283968 | 424 | CNPV294 ankyrin repeat protein |  |
| SWPV1-269 | 284004-284420 | 138 | CNPV186 IMV envelope protein |  |
| SWPV1-270 | 284463-285455 | 330 | CNPV131 conserved hypothetical protein |  |
| SWPV1-271 | 285608-286402 | 264 | CNPV295 ankyrin repeat protein |  |
| SWPV1-272 | 286454-287770 | 438 | CNPV296 ankyrin repeat protein |  |
| SWPV1-273 | 287864-290017 | 717 | CNPV297 ankyrin repeat protein |  |
| SWPV1-274 | 290053-291774 | 573 | CNPV298 ankyrin repeat protein |  |
| SWPV1-275 | 291784-292695 | 303 | CNPV299 putative serine/threonine protein kinase |  |
| SWPV1-276 | 292778-293539 | 253 | CNPV300 ankyrin repeat protein |  |
| SWPV1-277 | 293595-294023 | 142 | CNPV219 N1R/p28-like protein |  |
| SWPV1-278 | 294260-294523 | 87 | CNPV228 N1R/p28-like protein |  |
| SWPV1-279 | 294643-295941 | 432 | CNPV303 ankyrin repeat protein |  |
| SWPV1-280 | 295998-297530 | 510 | CNPV301 ankyrin repeat protein |  |
| SWPV1-281 | 297544-298071 | 175 | CNPV302 conserved hypothetical protein |  |
| SWPV1-282 | 298162-299661 | 499 | CNPV303 ankyrin repeat protein |  |
| SWPV1-283 | 299774-301204 | 476 | CNPV304 ankyrin repeat protein |  |
| SWPV1-284 | 301261-302046 | 261 | CNPV305 N1R/p28-like protein |  |
| SWPV1-285 | 302120-302323 | 67 | CNPV306 hypothetical protein |  |
| SWPV1-286 | 302320-302817 | 165 | CNPV307 C-type lectin-like protein |  |
| SWPV1-287 | 302994-304073 | 359 | CNPV308 ankyrin repeat protein |  |
| SWPV1-288 | 304177-304764 | 195 | CNPV309 ankyrin repeat protein |  |
| SWPV1-289 | 304812-306434 | 540 | CNPV310 ankyrin repeat protein |  |
| SWPV1-290 | 306444-306821 | 125 | CNPV311 EFc-like protein |  |
| SWPV1-291 | 306836-307342 | 168 | CNPV312 conserved hypothetical protein |  |
| SWPV1-292 | 307380-308021 | 213 | CNPV313 Ig-like domain protein |  |
| SWPV1-293 | 308049-309929 | 626 | CNPV314 ankyrin repeat protein |  |
| SWPV1-294 | 309994-311535 | 513 | CNPV021 ankyrin repeat protein |  |
| SWPV1-295 | 311590-312537 | 315 | CNPV315 G protein-coupled receptor-like protein |  |
| SWPV1-296 | 312693-313385 | 230 | CNPV014 Ig-like domain protein |  |
| SWPV1-297 | 313437-314159 | 240 | CNPV014 Ig-like domain protein |  |
| SWPV1-298 | 314309-314533 | 74 | CNPV015 ankyrin repeat protein |  |
| SWPV1-299 | 314551-314805 | 84 | CNPV150 ankyrin repeat protein |  |
| SWPV1-300 | 314811-315299 | 162 | CNPV316 ankyrin repeat protein |  |
| SWPV1-301 | 315391-315771 | 126 | CNPV321 EFc-like protein |  |
| SWPV1-302 | 315884-317575 | 563 | CNPV015 ankyrin repeat protein |  |
| SWPV1-303 | 317789-319231 | 480 | CNPV223 ankyrin repeat protein |  |
| SWPV1-304 | 319372-320778 | 468 | CNPV320 Ig-like domain protein |  |
| SWPV1-305 | 321407-321823 | 138 | CNPV035 C-type lectin-like protein |  |
| SWPV1-306 | 322000-322524 | 174 | CNPV008 C-type lectin-like protein |  |
| SWPV1-307 | 322653-322907 | 84 | CNPV323 conserved hypothetical protein |  |
| SWPV1-308 | 323041-323703 | 220 | CNPV324 conserved hypothetical protein |  |
| SWPV1-309 | 323829-325235 | 468 | CNPV325 ankyrin repeat protein |  |
| SWPV1-310 | 325747-326292 | 181 | CNPV326 C-type lectin-like protein |  |
