# Supplementary material for: Genomic characterization of two novel pathogenic avipoxviruses isolated from pacific shearwaters (Ardenna spp.)
Source: BMC Genomics. 2017 Apr 13;18:298. doi: 10.1186/s12864-017-3680-z (PMC5390406; doi:10.1186/s12864-017-3680-z)
Supplement: Supplementary file 2 — Summary of SWPV2 genome annotations (DOCX 145 kb) [file 12864_2017_3680_MOESM2_ESM.docx]

**Table S2** Summary of Shearwaterpox virus-2 (SWPV2) genome annotations

| **SWPV2 Annotation Table using CNPV as reference** | | | | |
| --- | --- | --- | --- | --- |
| **Name of SWPV gene** | **ORF position** | **#AA** | **Ortholog (according to CDS)** | **Annotation special notes** |
| SWPV2-001 | 1753-2268 | 171 | CNPV002 hypothetical protein |  |
| SWPV2-002 | 3226-2600 | 208 | CNPV003 C-type lectin-like protein |  |
| SWPV2-003 | 3641-4309 | 222 | CNPV005 conserved hypothetical protein |  |
| SWPV2-004 | 4426-4830 | 134 | CNPV006 hypothetical protein | Fragment |
| SWPV2-005 | 6376-5867 | 169 | CNPV008 C-type lectin-like protein |  |
| SWPV2-006 | 8730-6664 | 688 | CNPV009 ankyrin repeat protein |  |
| SWPV2-007 | 12177-10417 | 586 | CNPV011 ankyrin repeat protein |  |
| SWPV2-008 | 12442-13011 | 189 | CNPV012 hypothetical protein |  |
| SWPV2-009 | 13724-13218 | 168 | CNPV013 hypothetical protein |  |
| SWPV2-010 | 15514-14042 | 490 | CNPV014 immunoglobulin-like domain protein |  |
| SWPV2-011 | 15678-17264 | 528 | CNPV015 ankyrin repeat protein |  |
| SWPV2-012 | 17325-17831 | 168 | CNPV016 C-type lectin-like protein |  |
| SWPV2-013 | 17935-19374 | 479 | CNPV017 ankyrin repeat protein |  |
| SWPV2-014 | 20045-19473 | 190 | CNPV018 IL-10-like protein |  |
| SWPV2-015 | 21472-20162 | 436 | CNPV019 ankyrin repeat protein |  |
| SWPV2-016 | 21652-22911 | 419 | CNPV020 ankyrin repeat protein |  |
| SWPV2-017 | 24654-23047 | 535 | CNPV021 ankyrin repeat protein |  |
| SWPV2-018 | 25771-24695 | 358 | CNPV022 putative serpin |  |
| SWPV2-019 | 27127-25853 | 424 | CNPV023 vaccinia C4L/C10L-like protein |  |
| SWPV2-020 | 27405-27941 | 178 | CNPV024 hypothetical protein |  |
| SWPV2-021 | 29057-28155 | 300 | CNPV025 alpha-SNAP-like protein |  |
| SWPV2-022 | 30297-29149 | 382 | CNPV026 ankyrin repeat protein |  |
| SWPV2-023 | 32246-30366 | 626 | CNPV027 ankyrin repeat protein |  |
| SWPV2-024 | 33462-32365 | 365 | CNPV028 ankyrin repeat protein |  |
| SWPV2-025 | 34000-33572 | 142 | CNPV029 C-type lectin-like protein |  |
| SWPV2-026 | 35088-34066 | 340 | CNPV030 ankyrin repeat protein |  |
| SWPV2-027 | 35314-35673 | 119 | CNPV031 hypothetical protein |  |
| SWPV2-028 | 36629-35901 | 242 | CNPV032 Ig-like domain putative IFN-gamma binding protein |  |
| SWPV2-029 | 37445-36705 | 246 | CNPV033 Ig-like domain protein |  |
| SWPV2-030 | 39520-37541 | 659 | CNPV034 ankyrin repeat protein |  |
| SWPV2-031 | 40236-39835 | 133 | CNPV035 C-type lectin-like protein |  |
| SWPV2-032 | 40620-40333 | 95 | CNPV036 conserved hypothetical protein |  |
| SWPV2-033 | 40684-41223 | 179 | CNPV037 conserved hypothetical protein |  |
| SWPV2-034 | 42469-41228 | 413 | CNPV038 vaccinia C4L/C10L-like protein |  |
| SWPV2-035 | 42587-43570 | 327 | CNPV039 G protein-coupled receptor-like protein |  |
| SWPV2-036 | 45364-43589 | 591 | CNPV040 ankyrin repeat protein | High SNP density |
| SWPV2-037 | 46729-45437 | 430 | CNPV041 ankyrin repeat protein |  |
| SWPV2-038 | 48595-46778 | 605 | CNPV042 ankyrin repeat protein |  |
| SWPV2-039 | 49306-48701 | 201 | CNPV043 conserved hypothetical protein |  |
| SWPV2-040 | 50790-49348 | 480 | CNPV044 ankyrin repeat protein |  |
| SWPV2-041 | 51056-52054 | 332 | CNPV045 G protein-coupled receptor-like protein |  |
| SWPV2-042 | 53433-52081 | 450 | CNPV046 ankyrin repeat protein |  |
| SWPV2-043 | 53874-53500 | 124 | CNPV047 conserved hypothetical protein |  |
| SWPV2-044 | 56447-54042 | 801 | CNPV048 alkaline phosphodiesterase-like protein |  |
| SWPV2-045 | 56987-56535 | 150 | CNPV049 hypothetical protein |  |
| SWPV2-046 | 58099-57041 | 352 | CNPV050 ankyrin repeat protein |  |
| SWPV2-047 | 59372-58146 | 408 | CNPV051 DNase II-like protein |  |
| SWPV2-048 | 59913-59398 | 171 | CNPV052 C-type lectin-like protein |  |
| SWPV2-049 | 60533-60093 | 146 | CNPV053 conserved hypothetical protein |  |
| SWPV2-050 | 60948-60526 | 140 | CNPV054 conserved hypothetical protein |  |
| SWPV2-051 | 61491-61000 | 163 | CNPV055 conserved hypothetical protein |  |
| SWPV2-052 | 61925-61488 | 145 | CNLV056 dUTPase |  |
| SWPV2-053 | 62872-61952 | 306 | CNPV057 putative serpin |  |
| SWPV2-054 | 63445-62903 | 180 | CNPV058 bcl-2 like protein |  |
| SWPV2-055 | 64518-63502 | 338 | CNPV059 putative serpin |  |
| SWPV2-056 | 65202-64582 | 206 | CNPV060 conserved hypothetical protein | Fragment |
| SWPV2-057 | 66989-65292 | 565 | CNPV061 DNA ligase |  |
| SWPV2-058 | 68080-67028 | 350 | CNPV062 putative serpin |  |
| SWPV2-059 | 69227-68151 | 358 | CNPV063 hydroxysteroid dehydrogenase-like protein |  |
| SWPV2-060 | 70140-69289 | 283 | CNPV064 TGF-beta-like protein |  |
| SWPV2-061 | 71971-70220 | 583 | CNPV065 semaphorin-like protein |  |
| SWPV2-062 | 73270-72071 | 399 | CNPV066 hypothetical protein |  |
| SWPV2-063 | 73525-73352 | 57 | CNPV067 hypothetical protein |  |
| SWPV2-064 | 73685-74458 | 257 | CNPV068 GNS1/SUR4-like protein |  |
| SWPV2-065 | 74551-75018 | 155 | CNPV069 late transcription factor VLTF-2 |  |
| SWPV2-066 | 75035-76690 | 551 | CNPV070 putative rifampicin resistance protein, IMV assembly |  |
| SWPV2-067 | 76722-77591 | 289 | CNPV071 mRNA capping enzyme small subunit |  |
| SWPV2-068 | 77612-78010 | 132 | CNPV072 CC chemokine-like protein | Fragment |
| SWPV2-069 | 78952-78623 | 109 | CNPV073 hypothetical protein |  |
| SWPV2-070 | 79023-80930 | 635 | CNPV074 NPH-I, transcription termination factor |  |
| SWPV2-071 | 81613-80927 | 228 | CNPV075 mutT motif putative gene expression regulator |  |
| SWPV2-072 | 82295-81597 | 232 | CNPV076 mutT motif |  |
| SWPV2-073 | 84390-83908 | 160 | CNPV078 RNA polymerase subunit RPO18 |  |
| SWPV2-074 | 85550-84726 | 274 | CNPV079 Ig-like domain protein |  |
| SWPV2-075 | 87574-85673 | 633 | CNPV080 early transcription factor small subunit VETFS |  |
| SWPV2-076 | 88760-87756 | 334 | CNPV081 Ig-like domain protein |  |
| SWPV2-077 | 91438-89054 | 794 | CNPV082 NTPase, DNA replication |  |
| SWPV2-078 | 92258-91593 | 221 | CNPV083 CC chemokine-like protein |  |
| SWPV2-079 | 92997-92341 | 218 | CNPV084 uracil DNA glycosylase |  |
| SWPV2-080 | 94248-93337 | 303 | CNPV085 putative RNA phosphatase | High SNP density |
| SWPV2-081 | 94247-94585 | 112 | CNPV086 TNFR-like protein |  |
| SWPV2-082 | 94811-95206 | 131 | CNPV087 putative glutathione peroxidase | Fragment |
| SWPV2-083 | 95234-95536 | 100 | CNPV088 conserved hypothetical protein |  |
| SWPV2-084 | 96020-95541 | 159 | CNPV089 conserved hypothetical protein |  |
| SWPV2-085 | 96390-96007 | 127 | CNPV090 conserved hypothetical protein |  |
| SWPV2-086 | 96727-96476 | 83 | CNPV091 HT motif protein |  |
| SWPV2-087 | 97539-97099 | 146 | CNPV092 conserved hypothetical protein |  |
| SWPV2-088 | 98444-97641 | 267 | CNPV093 virion protein |  |
| SWPV2-089 | 98519-99346 | 275 | CNPV094 T10-like protein |  |
| SWPV2-090 | 99491-99354 | 45 | CNPV095 conserved hypothetical protein |  |
| SWPV2-091 | 99730-99473 | 85 | CNPV096 ubiquitin |  |
| SWPV2-092 | 100857-99838 | 339 | CNPV097 conserved hypothetical protein |  |
| SWPV2-093 | 101120-100878 | 80 | CNPV098 hypothetical protein |  |
| SWPV2-094 | 101713-101126 | 195 | CNPV099 beta-NGF-like protein |  |
| SWPV2-095 | 102243-101737 | 168 | CNPV100 putative interleukin binding protein |  |
| SWPV2-096 | 102555-102298 | 85 | CNPV101 hypothetical protein |  |
| SWPV2-097 | 102883-102566 | 105 | CNPV102 conserved hypothetical protein |  |
| SWPV2-098 | 103472-102900 | 190 | CNPV103 N1R/p28-like protein |  |
| SWPV2-099 | 103674-104051 | 125 | CNPV104 putative glutaredoxin 2, virion morphogenesis |  |
| SWPV2-100 | 104698-103994 | 234 | CNPV105 conserved hypothetical protein |  |
| SWPV2-101 | 104692-105000 | 102 | CNPV106 putative elongation factor |  |
| SWPV2-102 | 105136-105369 | 77 | CNPV107 hypothetical protein |  |
| SWPV2-103 | 105614-107512 | 632 | CNPV108 putative metalloprotease, virion morphogenesis |  |
| SWPV2-104 | 109541-107496 | 681 | CNPV109 NPH-II, RNA helicase |  |
| SWPV2-105 | 109576-110844 | 422 | CNPV110 virion core proteinase |  |
| SWPV2-106 | 110849-112024 | 391 | CNPV111 DNA-binding protein |  |
| SWPV2-107 | 112025-112270 | 81 | CNPV112 putative IMV membrane protein |  |
| SWPV2-108 | 112292-112831 | 179 | CNPV113 thymidine kinase |  |
| SWPV2-109 | 112952-113200 | 82 | CNPV114 HT motif protein |  |
| SWPV2-110 | 113270-114139 | 289 | CNPV115 DNA-binding phosphoprotein | High SNP density |
| SWPV2-111 | 114140-114349 | 69 | CNPV116 unnamed protein product |  |
| SWPV2-112 | 114356-115288 | 310 | CNPV117 DNA-binding virion protein |  |
| SWPV2-113 | 115468-117426 | 652 | CNPV118 conserved hypothetical protein |  |
| SWPV2-114 | 117356-117751 | 131 | CNPV119 virion core protein |  |
| SWPV2-115 | 118029-117748 | 93 | CNPV120 putative IMV redox protein, virus assembly |  |
| SWPV2-116 | 118056-121022 | 988 | CNPV121 DNA polymerase |  |
| SWPV2-117 | 123385-121877 | 502 | CNPV123 conserved hypothetical protein | High SNP density |
| SWPV2-118 | 129197-123447 | 1916 | CNPV124 variola B22R-like protein |  |
| SWPV2-119 | 134567-129264 | 1767 | CNPV125 variola B22R-like protein |  |
| SWPV2-120 | 140366-134847 | 1839 | CNPV126 variola B22R-like protein | Fragment |
| SWPV2-121 | 140688-140227 | 153 | CNPV126 variola B22R-like protein | Fragment |
| SWPV2-122 | 140778-141326 | 182 | CNPV127 RNA polymerase subunit RPO30 |  |
| SWPV2-123 | 141358-143523 | 721 | CNPV128 conserved hypothetical protein |  |
| SWPV2-124 | 143516-144934 | 472 | CNPV129 poly(A) polymerase large subunit PAPL |  |
| SWPV2-125 | 145287-144928 | 119 | CNPV130 DNA-binding virion core protein |  |
| SWPV2-126 | 145363-145986 | 207 | CNPV131 conserved hypothetical protein |  |
| SWPV2-127 | 146080-146526 | 148 | CNPV132 conserved hypothetical protein |  |
| SWPV2-128 | 146760-147059 | 99 | CNPV133 conserved hypothetical protein |  |
| SWPV2-129 | 152535-147130 | 1801 | CNPV134 variola B22R-like protein |  |
| SWPV2-130 | 152695-153831 | 378 | CNPV135 putative palmitylated EEV envelope lipase |  |
| SWPV2-131 | 153909-155786 | 625 | CNPV136 putative EEV maturation protein |  |
| SWPV2-132 | 155829-157217 | 462 | CNPV137 conserved hypothetical protein |  |
| SWPV2-133 | 157308-158642 | 444 | CNPV138 putative serine/threonine protein kinase, virus assembly |  |
| SWPV2-134 | 158617-159258 | 213 | CNPV139 conserved hypothetical protein |  |
| SWPV2-135 | 159341-159541 | 66 | CNPV140 conserved hypothetical protein |  |
| SWPV2-136 | 159867-160421 | 184 | CNPV141 HAL3-like domain protein |  |
| SWPV2-137 | 160682-161647 | 321 | CNPV142 N1R/p28-like protein |  |
| SWPV2-138 | 161759-163774 | 671 | CNPV143 ankyrin repeat protein |  |
| SWPV2-139 | 163800-165470 | 556 | CNPV144 ankyrin repeat protein |  |
| SWPV2-140 | 165691-167013 | 440 | CNPV145 conserved hypothetical protein |  |
| SWPV2-141 | 167021-167209 | 62 | CNPV146 RNA polymerase subunit RPO7 |  |
| SWPV2-142 | 167202-167768 | 188 | CNPV147 conserved hypothetical protein |  |
| SWPV2-143 | 168779-167733 | 348 | CNPV148 virion core protein |  |
| SWPV2-144 | 169865-168945 | 306 | CNPV149 putative thioredoxin binding protein |  |
| SWPV2-145 | 171599-170361 | 412 | CNPV151 ankyrin repeat protein |  |
| SWPV2-146 | 172275-171826 | 149 | CNPV152 hypothetical protein | Fragment |
| SWPV2-147 | 173441-172503 | 312 | CNPV153 Rep-like protein |  |
| SWPV2-148 | 179653-177026 | 875 | CNPV154 variola B22R-like protein | Fragment |
| SWPV2-149 | 185201-179706 | 1831 | CNPV155 variola B22R-like protein |  |
| SWPV2-150 | 185518-188022 | 834 | CNPV156 hypothetical protein |  |
| SWPV2-151 | 189150-188119 | 343 | CNPV157 TGF-beta-like protein |  |
| SWPV2-152 | 193047-191971 | 358 | CNPV161 TGF-beta-like protein |  |
| SWPV2-153 | 193097-193546 | 149 | CNPV162 TGF-beta-like protein |  |
| SWPV2-154 | 193964-194926 | 320 | CNPV165 N1R/p28-like protein | Fragment |
| SWPV2-155 | 195161-196198 | 345 | CNPV166 Ig-like domain protein |  |
| SWPV2-156 | 196467-196973 | 168 | CNPV167 Ig-like domain protein |  |
| SWPV2-157 | 197068-198120 | 350 | CNPV168 N1R/p28-like protein |  |
| SWPV2-158 | 199162-199800 | 212 | CNPV170 thymidylate kinase |  |
| SWPV2-159 | 199853-200635 | 260 | CNPV171 late transcription factor VLTF-1 |  |
| SWPV2-160 | 200649-201656 | 335 | CNPV172 putative myristylated protein |  |
| SWPV2-161 | 201657-202388 | 243 | CNPV173 putative myristylated IMV envelope protein |  |
| SWPV2-162 | 202448-202738 | 96 | CNPV174 conserved hypothetical protein |  |
| SWPV2-163 | 203639-202728 | 303 | CNPV175 conserved hypothetical protein |  |
| SWPV2-164 | 203665-204423 | 252 | CNPV176 DNA-binding virion core protein |  |
| SWPV2-165 | 204424-204816 | 130 | CNPV177 conserved hypothetical protein |  |
| SWPV2-166 | 204770-205216 | 148 | CNPV178 putative IMV membrane protein |  |
| SWPV2-167 | 205250-206158 | 302 | CNPV179 poly(A) polymerase small subunit PAPS |  |
| SWPV2-168 | 206155-206715 | 186 | CNPV180 RNA polymerase subunit RPO22 |  |
| SWPV2-169 | 207118-206708 | 136 | CNPV181 conserved hypothetical protein |  |
| SWPV2-170 | 207161-211027 | 1288 | CNPV182 RNA polymerase subunit RPO147 |  |
| SWPV2-171 | 211530-211030 | 166 | CNPV183 putative protein-tyrosine phosphatase, virus assembly |  |
| SWPV2-172 | 211546-212115 | 189 | CNPV184 conserved hypothetical protein |  |
| SWPV2-173 | 213177-212191 | 328 | CNPV185 ankyrin repeat protein |  |
| SWPV2-174 | 214212-213220 | 330 | CNPV186 IMV envelope protein |  |
| SWPV2-175 | 216703-214304 | 799 | CNPV187 RNA polymerase associated protein RAP94 |  |
| SWPV2-176 | 216872-217384 | 170 | CNPV188 late transcription factor VLTF-4 |  |
| SWPV2-177 | 217385-218335 | 316 | CNPV189 DNA topoisomerase |  |
| SWPV2-178 | 218340-218801 | 153 | CNPV190 conserved hypothetical protein |  |
| SWPV2-179 | 219075-218764 | 103 | CNPV191 conserved hypothetical protein |  |
| SWPV2-180 | 219083-221623 | 846 | CNPV192 mRNA capping enzyme large subunit |  |
| SWPV2-181 | 221694-222014 | 106 | CNPV193 HT motif protein |  |
| SWPV2-182 | 222433-222011 | 140 | CNPV194 virion protein |  |
| SWPV2-183 | 222487-222921 | 144 | CNPV195 hypothetical protein |  |
| SWPV2-184 | 222986-223558 | 190 | CNPV196 conserved hypothetical protein |  |
| SWPV2-185 | 223624-224451 | 275 | CNPV197 N1R/p28-like protein |  |
| SWPV2-186 | 224988-224518 | 156 | CNPV198 C-type lectin-like protein |  |
| SWPV2-187 | 225296-225973 | 225 | CNPV199 deoxycytidine kinase-like protein |  |
| SWPV2-188 | 225979-226479 | 166 | CNPV200 Rep-like protein |  |
| SWPV2-189 | 226538-227041 | 167 | CNPV201 conserved hypothetical protein |  |
| SWPV2-190 | 227095-227925 | 276 | CNPV202 N1R/p28-like protein |  |
| SWPV2-191 | 227998-229146 | 382 | CNPV203 N1R/p28-like protein |  |
| SWPV2-192 | 229202-229387 | 61 | CNPV204 conserved hypothetical protein |  |
| SWPV2-193 | 229606-230562 | 318 | CNPV205 N1R/p28-like protein |  |
| SWPV2-194 | 230623-232041 | 472 | CNPV206 putative photolyase |  |
| SWPV2-195 | 232170-232691 | 173 | CNPV207 N1R/p28-like protein |  |
| SWPV2-196 | 232860-233462 | 200 | CNPV208 conserved hypothetical protein |  |
| SWPV2-197 | 233506-234438 | 310 | CNPV209 N1R/p28-like protein |  |
| SWPV2-198 | 234486-234881 | 131 | CNPV210 N1R/p28-like protein |  |
| SWPV2-199 | 234936-235100 | 54 | CNPV211 conserved hypothetical protein |  |
| SWPV2-200 | 235160-235690 | 176 | CNPV212 N1R/p28-like protein |  |
| SWPV2-201 | 236384-235734 | 216 | CNPV213 deoxycytidine kinase-like protein |  |
| SWPV2-202 | 236558-237628 | 356 | CNPV214 vaccinia C4L/C10L-like protein |  |
| SWPV2-203 | 237903-238517 | 204 | CNPV215 CC chemokine-like protein |  |
| SWPV2-204 | 238607-239812 | 401 | CNPV216 conserved hypothetical protein |  |
| SWPV2-205 | 239907-240899 | 330 | CNPV217 N1R/p28-like protein |  |
| SWPV2-206 | 240987-241658 | 223 | CNPV218 N1R/p28-like protein | Fragment |
| SWPV2-207 | 243116-244165 | 349 | CNPV219 N1R/p28-like protein |  |
| SWPV2-208 | 244219-244476 | 85 | CNPV220 N1R/p28-like protein | Fragment |
| SWPV2-209 | 244753-245394 | 213 | CNPV221 N1R/p28-like protein | Fragment |
| SWPV2-210 | 245862-246719 | 285 | CNPV222 N1R/p28-like protein |  |
| SWPV2-211 | 249781-247238 | 847 | CNPV223 ankyrin repeat protein |  |
| SWPV2-212 | 250035-250754 | 239 | CNPV224 hypothetical protein |  |
| SWPV2-213 | 250829-251113 | 94 | CNPV225 N1R/p28-like protein | Fragment |
| SWPV2-214 | 251328-251708 | 126 | CNPV226 N1R/p28-like protein |  |
| SWPV2-215 | 255478-254174 | 434 | CNPV229 ankyrin repeat protein |  |
| SWPV2-216 | 255676-255873 | 65 | CNPV230 hypothetical protein |  |
| SWPV2-217 | 255821-256297 | 158 | CNPV231 MyD116-like domain protein |  |
| SWPV2-218 | 256327-256941 | 204 | CNPV232 CC chemokine-like protein |  |
| SWPV2-219 | 257081-258496 | 471 | CNPV233 ankyrin repeat protein |  |
| SWPV2-220 | 258516-260042 | 508 | CNPV234 ankyrin repeat protein | High SNP density |
| SWPV2-221 | 260113-261411 | 432 | CNPV235 conserved hypothetical protein |  |
| SWPV2-222 | 261456-262427 | 323 | CNPV236 ribonucleotide reductase small subunit |  |
| SWPV2-223 | 262608-263933 | 441 | CNPV237 ankyrin repeat protein |  |
| SWPV2-224 | 264649-263972 | 225 | CNPV238 late transcription factor VLTF-3 |  |
| SWPV2-225 | 264864-264637 | 75 | CNPV239 virion redox protein |  |
| SWPV2-226 | 266857-264878 | 659 | CNPV240 virion core protein P4b |  |
| SWPV2-227 | 267591-266944 | 215 | CNPV241 immunodominant virion protein |  |
| SWPV2-228 | 267630-268139 | 169 | CNPV242 RNA polymerase subunit RPO19 |  |
| SWPV2-229 | 269255-268134 | 373 | CNPV243 conserved hypothetical protein |  |
| SWPV2-230 | 271391-269262 | 709 | CNPV244 early transcription factor large subunit VETFL |  |
| SWPV2-231 | 271455-272357 | 300 | CNPV245 intermediate transcription factor VITF-3 |  |
| SWPV2-232 | 272549-272322 | 75 | CNPV246 putative IMV membrane protein |  |
| SWPV2-233 | 275231-272550 | 893 | CNPV247 virion core protein P4a |  |
| SWPV2-234 | 275249-276088 | 279 | CNPV248 conserved hypothetical protein |  |
| SWPV2-235 | 276591-276085 | 168 | CNPV249 virion protein |  |
| SWPV2-236 | 276606-276776 | 56 | CNPV250 conserved hypothetical protein | Fragment |
| SWPV2-237 | 277099-276890 | 69 | CNPV251 putative IMV membrane protein |  |
| SWPV2-238 | 277425-277147 | 92 | CNPV252 putative IMV membrane protein |  |
| SWPV2-239 | 277603-277442 | 53 | CNPV253 putative IMV membrane virulence factor |  |
| SWPV2-240 | 277909-277619 | 96 | CNPV254 conserved hypothetical protein |  |
| SWPV2-241 | 278999-277893 | 368 | CNPV255 predicted myristylated protein |  |
| SWPV2-242 | 279593-279015 | 192 | CNPV256 putative phosphorylated IMV membrane protein |  |
| SWPV2-243 | 279611-280999 | 462 | CNPV257 DNA helicase, transcriptional elongation |  |
| SWPV2-244 | 281236-280967 | 89 | CNPV258 conserved hypothetical protein |  |
| SWPV2-245 | 281582-281244 | 112 | CNPV259 DNA polymerase processivity factor |  |
| SWPV2-246 | 281581-282885 | 434 | CNPV260 conserved hypothetical protein |  |
| SWPV2-247 | 282882-283340 | 152 | CNPV261 Holliday junction resolvase protein |  |
| SWPV2-248 | 283357-284508 | 383 | CNPV262 intermediate transcription factor VITF-3 |  |
| SWPV2-249 | 284534-288007 | 1157 | CNPV263 RNA polymerase subunit RPO132 |  |
| SWPV2-250 | 289801-287996 | 601 | CNPV264 A type inclusion-like protein |  |
| SWPV2-251 | 291263-289836 | 475 | CNPV265 A type inclusion-like/fusion protein |  |
| SWPV2-252 | 291686-291264 | 140 | CNPV266 conserved hypothetical protein |  |
| SWPV2-253 | 292608-291691 | 305 | CNPV267 RNA polymerase subunit RPO35 |  |
| SWPV2-254 | 292810-292583 | 75 | CNPV268 conserved hypothetical protein |  |
| SWPV2-255 | 292935-293276 | 113 | CNPV269 conserved hypothetical protein |  |
| SWPV2-256 | 293285-293647 | 120 | CNPV270 conserved hypothetical protein |  |
| SWPV2-257 | 294490-293636 | 284 | CNPV271 DNA packaging protein |  |
| SWPV2-258 | 294605-295150 | 181 | CNPV272 C-type lectin-like EEV protein |  |
| SWPV2-259 | 295375-296199 | 274 | CNPV273 conserved hypothetical protein |  |
| SWPV2-260 | 296259-297068 | 269 | CNPV274 putative tyrosine protein kinase |  |
| SWPV2-261 | 297111-298127 | 338 | CNPV275 putative serpin |  |
| SWPV2-262 | 298907-298149 | 252 | CNPV276 conserved hypothetical protein |  |
| SWPV2-263 | 299017-299949 | 310 | CNPV277 G protein-coupled receptor-like protein |  |
| SWPV2-264 | 299960-300250 | 96 | CNPV278 conserved hypothetical protein |  |
| SWPV2-265 | 300316-300825 | 169 | CNPV279 beta-NGF-like protein |  |
| SWPV2-266 | 301235-300843 | 130 | CNPV280 HT motif protein |  |
| SWPV2-267 | 301339-301983 | 214 | CNPV281 conserved hypothetical protein |  |
| SWPV2-268 | 302356-301994 | 120 | CNPV282 HT motif protein |  |
| SWPV2-269 | 302522-302857 | 111 | CNPV283 CC chemokine-like protein |  |
| SWPV2-270 | 302929-303510 | 193 | CNPV284 putative interleukin binding protein |  |
| SWPV2-271 | 303620-304000 | 126 | CNPV285 EGF-like protein |  |
| SWPV2-272 | 304002-304919 | 305 | CNPV286 putative serine/threonine protein kinase |  |
| SWPV2-273 | 304962-305444 | 160 | CNPV287 conserved hypothetical protein |  |
| SWPV2-274 | 305480-305923 | 147 | CNPV288 C-type lectin-like protein |  |
| SWPV2-275 | 305966-306385 | 139 | CNPV289 putative interleukin binding protein |  |
| SWPV2-276 | 306454-306681 | 75 | CNPV290 conserved hypothetical protein |  |
| SWPV2-277 | 306883-308667 | 594 | CNPV291 ankyrin repeat protein |  |
| SWPV2-278 | 308691-308915 | 74 | CNPV292 hypothetical protein |  |
| SWPV2-279 | 308958-309812 | 284 | CNPV293 ankyrin repeat protein |  |
| SWPV2-280 | 309867-311159 | 430 | CNPV294 ankyrin repeat protein |  |
| SWPV2-281 | 311352-312542 | 396 | CNPV295 ankyrin repeat protein |  |
| SWPV2-282 | 312545-313921 | 458 | CNPV296 ankyrin repeat protein |  |
| SWPV2-283 | 314018-316231 | 737 | CNPV297 ankyrin repeat protein |  |
| SWPV2-284 | 316287-318002 | 571 | CNPV298 ankyrin repeat protein |  |
| SWPV2-285 | 318006-318908 | 300 | CNPV299 putative serine/threonine protein kinase |  |
| SWPV2-286 | 318981-319715 | 244 | CNPV300 ankyrin repeat protein |  |
| SWPV2-287 | 320316-321899 | 527 | CNPV301 ankyrin repeat protein |  |
| SWPV2-288 | 322495-321914 | 193 | CNPV302 conserved hypothetical protein |  |
| SWPV2-289 | 322563-324065 | 500 | CNPV303 ankyrin repeat protein |  |
| SWPV2-290 | 324281-325681 | 466 | CNPV304 ankyrin repeat protein |  |
| SWPV2-291 | 325752-326540 | 262 | CNPV305 N1R/p28-like protein |  |
| SWPV2-292 | 326602-326820 | 72 | CNPV306 hypothetical protein |  |
| SWPV2-293 | 327288-326824 | 154 | CNPV307 C-type lectin-like protein |  |
| SWPV2-294 | 327465-328538 | 357 | CNPV308 ankyrin repeat protein |  |
| SWPV2-295 | 328686-329276 | 196 | CNPV309 ankyrin repeat protein |  |
| SWPV2-296 | 329381-330994 | 537 | CNPV310 ankyrin repeat protein |  |
| SWPV2-297 | 331028-331402 | 124 | CNPV311 EFc-like protein |  |
| SWPV2-298 | 331412-331912 | 166 | CNPV312 conserved hypothetical protein |  |
| SWPV2-299 | 331984-332640 | 218 | CNPV313 Ig-like domain protein |  |
| SWPV2-300 | 332667-334556 | 629 | CNPV314 ankyrin repeat protein |  |
| SWPV2-301 | 334655-335602 | 315 | CNPV315 G protein-coupled receptor-like protein |  |
| SWPV2-302 | 335669-337303 | 544 | CNPV316 ankyrin repeat protein |  |
| SWPV2-303 | 337484-337651 | 55 | CNPV317 hypothetical protein |  |
| SWPV2-304 | 337826-339370 | 514 | CNPV318 ankyrin repeat protein |  |
| SWPV2-305 | 339792-341705 | 637 | CNPV319 ankyrin repeat protein | Fragment |
| SWPV2-306 | 341896-343305 | 469 | CNPV320 Ig-like domain protein |  |
| SWPV2-307 | 343437-343811 | 124 | CNPV321 EFc-like protein |  |
| SWPV2-308 | 344145-346214 | 689 | CNPV322 ankyrin repeat protein |  |
| SWPV2-309 | 346806-346246 | 186 | CNPV323 conserved hypothetical protein |  |
| SWPV2-310 | 347434-346766 | 222 | CNPV324 conserved hypothetical protein |  |
| SWPV2-311 | 347852-348478 | 208 | CNPV326 C-type lectin-like protein |  |
| SWPV2-312 | 349319-348804 | 171 | CNPV327 hypothetical protein |  |
